# DNA structure at the plasmid origin-of-transfer indicates its potential transfer range

**DOI:** 10.1038/s41598-018-20157-y

**Published:** 2018-01-29

**Authors:** Jan Zrimec, Aleš Lapanje

**Affiliations:** 1 Institute of Metagenomics and Microbial Technologies, 1000 Ljubljana, Slovenia; 2 Faculty of Health Sciences, University of Primorska, 6320 Izola, Slovenia; 3 Department of Biology and Biological Engineering, Chalmers University of Technology, 412 96, Göteborg, Sweden; 4 Department of Nanotechnology, Saratov State University, 410012, Saratov, Russian Federation; 5 Department of Environmental Sciences, Institute Jožef Štefan, 1000 Ljubljana, Slovenia

## Abstract

Horizontal gene transfer via plasmid conjugation enables antimicrobial resistance (AMR) to spread among bacteria and is a major health concern. The range of potential transfer hosts of a particular conjugative plasmid is characterised by its mobility (MOB) group, which is currently determined based on the amino acid sequence of the plasmid-encoded relaxase. To facilitate prediction of plasmid MOB groups, we have developed a bioinformatic procedure based on analysis of the origin-of-transfer (*oriT*), a merely 230 bp long non-coding plasmid DNA region that is the enzymatic substrate for the relaxase. By computationally interpreting conformational and physicochemical properties of the *oriT* region, which facilitate relaxase-*oriT* recognition and initiation of nicking, MOB groups can be resolved with over 99% accuracy. We have shown that *oriT* structural properties are highly conserved and can be used to discriminate among MOB groups more efficiently than the *oriT* nucleotide sequence. The procedure for prediction of MOB groups and potential transfer range of plasmids was implemented using published data and is available at http://dnatools.eu/MOB/plasmid.html.

## Introduction

Antimicrobial resistance (AMR) is a pressing global issue, as it diminishes the activity of 29 antibiotics and consequently leads to over 25,000 deaths each year in Europe alone^1,2^. The development of AMR in microbial communities is facilitated by horizontal gene transfer (HGT) of conjugative elements (including plasmids and integrative elements)^3^ carrying antibiotic resistance genes along with virulence genes^4,5^. It is therefore important to determine the routes of plasmid transfer among bacteria^6,7^, based on determining their host range^8^.

**Figure 1:**
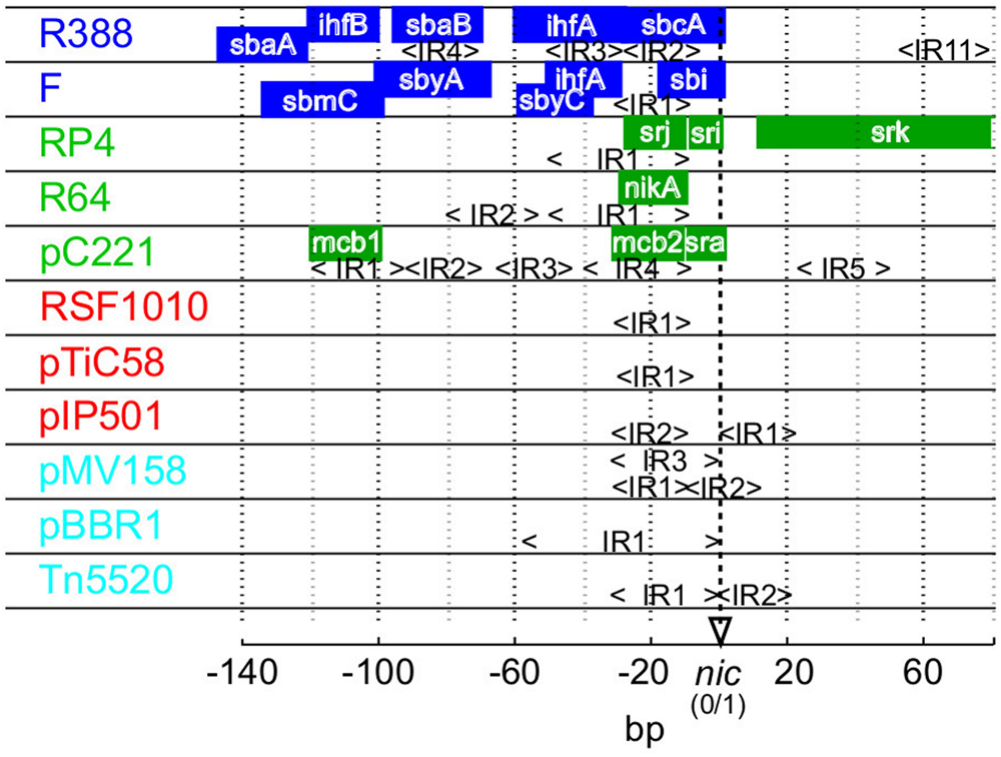
Schematic representation of available experimental data on *oriT* regions from four MOB groups. o*riT* data from MOB F (blue), P (green), Q (red) and V (cyan) supports that the conservation of structural properties within each MOB group is greater than between groups. Known binding sites for auxiliary proteins and relaxases are marked (colored squares) and are frequently characterized by inverted repeats (<IR>). Relaxase binding sites are nearest to the *nic* site (between 0 and 1 bp). General characteristics of MOB groups are: (F) system of multiple auxiliary proteins including the DNA-bending protein IHF^44,55^, (P) up to 5 proteins including relaxase involved in relaxation (RP4)^47,54,61^ (Q) a shorter *oriT* region of only 38 bp that covers besides relaxase 2 auxiliary proteins without clear binding sites (RSF1010)^48,49,62^, (V) no known auxiliary proteins^50,51,63^.

It is currently known that each of the 6 established mobility superclasses of conjugative elements have limited transfer host range^8^. Conjugation systems of each of these MOB groups are classified according to the conservation of the amino acid sequences of relaxase, the central enzyme that enables relaxation and transfer of elements from donor to recipient cells^9,10^. Besides relaxases, the relative conservative nature of MOB groups can be detected among other protein components of conjugation systems, which are comprised of (i) auxiliary proteins that take part in formation of the relaxation complex (relaxosome) in the origin of transfer (*oriT*) DNA region^11^, (ii) coupling protein (type IV)^12,13^, which connects the relaxosome with (iii) the mating complex (type IV secretion system, T4SS) that forms the transfer channel between donor and recipient cells^14^. These protein components were shown to coevolve to a large extent within their respective MOB groups^12,13,15^. In addition to the conservative nature of proteins involved in DNA transfer, it has also been observed that a relaxase from a certain MOB group enables the most efficient transfer only of plasmids belonging to that same group^16^. Therefore, one can expect that the substrate for relaxases, the bare noncoding sites in *oriT*, should also possess some MOB-specific properties that enable their cognate relaxases to initiate the conjugation process most efficiently (Fig. 1, Table 1).

**Table 1:** *oriT* structural properties that enable relaxasome formation and nicking of DNA to initiate transfer of conjugative elements. Shown are experimentally determined *oriT* structural features and predicted structural properties that were used to interpret them.

Experimental *oriT* structural features	Reference	Predicted properties
Direct and inverted repeats of DNA sequence around *nic* that define extensive secondary structures (e.g. hairpins) and act as protein-DNA recognition regions	^44,64^	Deformability *S*_*Def*_
		Duplex stability *S*_*Stab*_
DNA melting bubbles and destabilizations that facilitate relaxase nicking, aided by lower duplex stability around *nic* and by DNA thermal dynamics	^21,33,65,66^	Thermally induced duplex destabilizations *S*_*TIDD*_
Intrinsically curved or flexible regions that facilitate binding and changes in *oriT* structure around *nic* (e.g. IHF protein binding in MOB F)	^44,55,67^	Bending propensity *S*_*Bend*_
		Persistence length *S*_*Per*_
Differences in DNA spacing and orientation between binding sites and *nic*	^68^	Helical repeats *S*_*Hel*_

The specific conservation of *oriT* properties within MOB groups can also be expected, since DNA binding proteins recognize a particular site on DNA by a physicochemical interaction with the DNA. Prior to binding, proteins slide on DNA in controlled 1D diffusion processes in search of their active binding sites^17,18^. Therefore, some of the essential features of DNA recognition that optimize the protein-DNA indirect readout process are the conformational and physicochemical DNA structural properties at the specific binding sites and around them^19,20^. In the case of initiation of conjugation, the *oriT* region is a recognition site and it is also an enzymatic substrate, since the relaxase recognizes specific DNA as well as makes a nick in the DNA to initiate conjugation^21,22^. However, contrary to the conserved amino or nucleic acid sequences of relaxases and auxiliary proteins, the *oriT* is a noncoding region and low conservation of nucleotide sequence is expected^9,11^.

Therefore, in order to pinpoint the specific properties in *oriT* that are conserved within plasmids of a particular MOB group, the conventional approach based on clustering of similar DNA sequences is unlikely to be successful. A more advanced approach is required to classify MOB groups based on the analysis of *oriT* structural properties. The aims of the present study were to (i) analyze the DNA structural properties of *oriT* regions from different MOB groups (Fig. 1, Table 1), (ii) determine if DNA structural properties are conserved within MOB groups and can be used to discriminate among them and (iii) implement the classification procedure as a webtool available to the wider research community.

## Methods

### *oriT* datasets

To construct and analyze statistical and predictive models a training and a testing dataset were used. The training dataset comprised nucleotide sequences of *oriT* regions of 64 elements that were obtained from the Genbank database. In these sequences the o*riT* regions were identified and aligned according to published experimental information on *nic* sites (Supp. Table S1). Despite the scarce amount of published data, which limited the amount of MOB groups used and the size of the training dataset, the dataset was balanced, with approximately 16 elements from each MOB and contained *oriT*s from all known MOB subgroups^10^. For the testing dataset we obtained 136 *oriT* regions from plasmids, for which the only previously available information was that of their MOB groups, determined on the basis of amino acid sequences of relaxases^10^. The locations of *nic* sites in these plasmids were determined by finding the minimal Euclidean distance between structural properties of training *oriT*s and the testing dataset. The positions of resulting *oriT* regions were verified using experimental data and relaxase locations^10^ (Supp. Table S2). By combining the training and testing datasets, the expanded dataset of 200 elements was of an appropriate size to support a statistical and machine learning analysis (Supp. Fig. S1: see learning curves). The testing dataset was thus used for cross validations as well as training of predictive models. In both datasets the part of the *oriT* regions with relevant protein binding features from −140 bp to +80 bp according to the *nic* site were analysed (Fig. 1: see references).

### Nucleotide sequence analysis

**Figure 2:**
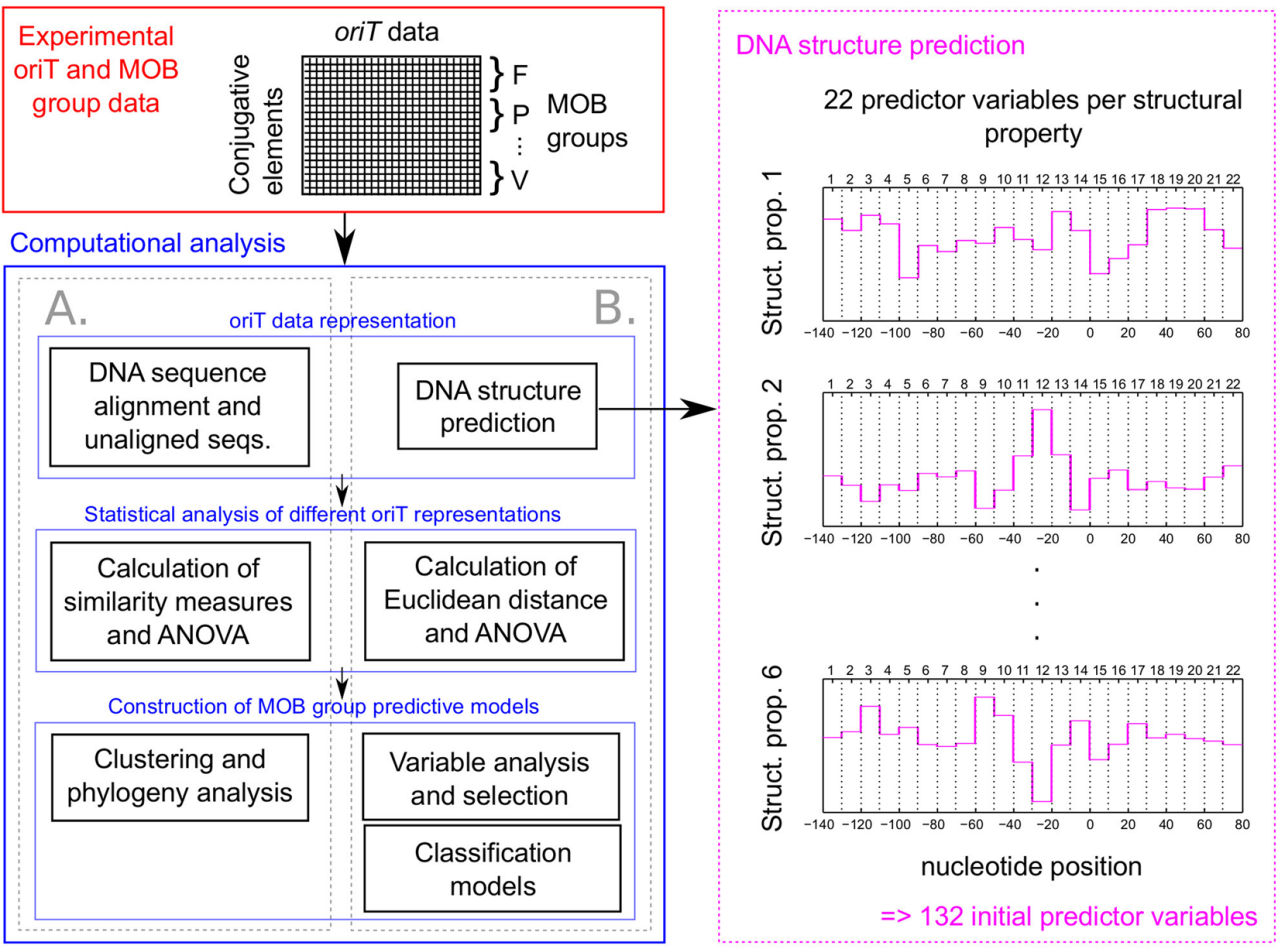
Overview of the performed computational analysis. DNA sequences of *oriT* regions and their MOB groups were used to compare (**A**) the conventional approach based on analysis of primary sequences with (**B**) our new approach based on DNA structure prediction. *oriT* regions were ligned up to the *nic* functional site.

The *oriT* dataset of 200 elements was aligned using the ClustalW algorithm^23^ and grouped based on the following distances between DNA sequences: (I) the p-distance: the ratio of the amount of different sequence positions to sequence size, and (ii) the 2-parameter Kimura distance: models transitional and transversional nucleotide substitution rates^24^ (Fig. 2). Clustering of similar sequences was performed with the Neighbor Joining method using p-distance, and with the Maximum Likelihood method using the Kimura distance. The topology of constructed trees was tested with the bootstrap^25^. The classification accuracy of condensed trees was estimated as the average ratio of branches that contained elements from a specific MOB group to all elements in that group. Mega version 6.06 software^26^ was used for all calculations with default settings. The bootstrap parameter was set to 1000 repetitions and cutoff values of 50% and 80% were used for positioning of branches within a constructed tree. DNA sequence conservation per basepair was evaluated using information content analysis based on Shannon’s entropy, where the maximum information content of 2 bits reflected maximum sequence conservation and vice-versa^27,28^.

### Prediction of structural variables

In contrast to the conventional sequence-based analysis, an alternative representation of *oriT* regions was developed based on computed DNA structural properties (Fig. 2). Parametric models were used to predict conformational and physicochemical properties. Conformational properties included (i) DNA deformability, which affects DNA-protein interactions, as given by volumes of conformation space (*S*_*Def*_) with the model based on data of DNA-protein crystal complexes^29^, (ii) DNA bending propensity (*S*_*Bend*_) with the model based on DNaseI enzyme digestion data^30^ and (iii) DNA persistence length (*S*_*Per*_, proportional to stiffness) and DNA helical repeats (*S*_*Hel*_, equal to number of bps per helix turn) with the model based on cyclization experiments of short DNA fragments^31^. Physicochemical properties included (i) relative DNA duplex stability (*S*_*Stab*_) with the thermodynamic nearest neighbor (NN) model using the unified NN parameters at 37 °C^32^ and (ii) thermally induced duplex destabilization (TIDD, *S*_*TIDD*_) with our recently developed method based on machine learning algorithms using 6 bp of neighboring regions at a threshold of 0.1 Å^33^. Predicted structural properties spanned 10 bp using a sliding window approach, due to its potential to detect the conserved regions among similar MOB groups with higher accuracy and solve the problem of leftover nucleotides at the end of the sequence. To increase the ratio of signal to noise, the predictions were averaged in windows of 10 consecutive basepairs (Fig. 2). This also decreased the number of variables used in the analysis per DNA structural property and per *oriT* region from an initial 220 to 22. For calculation of the DNA sequence and structural properties Matlab software (Mathworks, MA, USA) was used.

### Statistical analysis

A central measure of the conservation of data within groups is the ratio of the variability of the data between groups versus the average variability of data in each group, which is given as the *F* statistic and can be statistically evaluated with analysis of variance (ANOVA). Since our data did not follow a normal distribution (Supp. methods S1), a non-parametric multivariate ANOVA^34^ was used (Supp. Methods S2). In this procedure the variability of the data was evaluated based on an inter-point geometric approach that enabled the use of different distance measures including: (i) the p-distance with nucleotide sequences and (ii) the Euclidean distance with structural variables. The same non-parametric procedure was used to analyze the conservation of (i) individual structural variables and (ii) nucleotide sequences at specific *oriT* positions in windows of 10 bp (Fig. 2: comparison at 22 positions). To avoid Type I errors due to multiple comparisons the Bonferroni correction was applied^35^. Differences between means of groups of data were tested with the Mann-Whitney-Wilcoxon test^36^. Input data was standardized to zero mean and unit variance. All analyses were performed in Matlab, except distribution analysis for which SPSS ver. 22 (IBM, NY, USA) was used.

### Variable analysis and selection

Subsets of the most informative structural variables for predicting MOB groups were obtained using a backward variable selection procedure. The procedure included (i) ranking of variables according to one of three criteria of relative variable importance, and (ii) performing backward selection with classification tests, to select the optimal subset that led to highest classification measures (see ‘Construction of predictive models’ below). The initial criteria for ranking of variables were based on *p*-values of the *F* statistic. However, since the ANOVA procedure that was used did not enable analysis of potential interactions between variables, which were presumed to play an important role in discrimination between groups, two of the most efficient and frequently used variable selection algorithms^37^ were applied to detect interactions between variable. These were (i) Correlation-based feature selection (CFS) Subset Evaluator algorithm^38^ with the Greedy Stepwise search method to detect moderate levels of interaction and (ii) ReliefF Attribute Evaluator algorithm^39^ with the Ranker search method used to detect higher order interactions.

### Construction of predictive models

Two types of classification tests were performed using either (i) different subsets of predictor variables or (ii) different subsets of data. In the backward variable selection procedure, the influence of the number of ranked variables on MOB prediction was evaluated by stepwise removal of variables with the lowest ranks. With each subset, 10 repetitions of classification tests were performed. To evaluate the effect of removing elements with low classification frequency (the ratio of correct classifications to number of classifications) from the training dataset, 100 repetitions were performed. The classification tests comprised (i) 10-fold cross validations (CVs) using the training dataset (*CV_64*), (ii) 10-fold CVs using the full set of 200 elements (*CV_200*) and (iii) testing the trained models with the testing dataset (*Test*). The classification tests were evaluated with six of the most relevant classification performance measures for multi-group classification (Supp. Methods S3)^40–43^, including Precision (*Pre*) and Recall (*Rec*). The Multilayer perceptron algorithm with default settings was used for construction and testing of predictive models. Matlab was used to run the algorithms and to analyze the data. Algorithm implementations in Weka software^43^ version 3.7.9 were used.

## Results

### Structure prediction improves discrimination of MOB groups

The conventional phylogenetic sequence analysis of the dataset of *oriT* regions (Fig. 2, Supp. Tables S1 and S2) led to an inaccurate discrimination of MOB groups. Dendrograms of aligned *oriT* sequences based on calculated sequence distances, either p-distance or Kimura, contained large numbers of clusters (up to 48 per MOB group) from which elements could not be sorted into their respective MOB groups (Supp. Fig. S2A–D: estimated class. accuracy did not exceed 0.110 ± 0.104; 95% confidence bounds given) Therefore, a different sequence alignment approach was used, in which *oriT* sequences were lined up according to the *nic* site (see Table 1, Fig. 1). However, the results again indicated that MOB groups could not be correctly resolved (Supp. Fig. S2E–H: estimated class. accuracy did not exceed 0.082 ± 0.045). The *oriT* region also showed low information content, i.e. low sequence conservation in individual MOB groups (Supp. Fig. S3: below 0.518 bits) and even lower among all MOB groups (below 0.152 bits) both in sequence and *nic* based alignments. However, the *F* statistic obtained from the analysis of variance of MOB groups by comparing the overall variance of data between groups with the variance of data within groups was shown to be statistically significant with the aligned sequences at an alpha level of 0.05 (*F* = 0.728, *p* = 0.029), contrary to the *nic* based alignment (*F* = 0.525, *p* = 0.475).

Since the *oriT* region contains many structural features that were presumed to be crucial for achieving better MOB discrimination, we predicted 6 known structural properties as an alternative representation of *oriT* data (see Table 1 and Fig. 2). Using the structural variables a significantly larger F statistic was obtained than with unaligned and aligned sequences (*p* < 0.001 and *p* = 0.047, respectively), showing significantly higher conservation of structural properties within MOB groups (*F* = 1.000, *p* < 0.001; Supp. Table S3).

### Predicted structural properties distinguish functionally important sites in *oriT*

**Figure 3:**
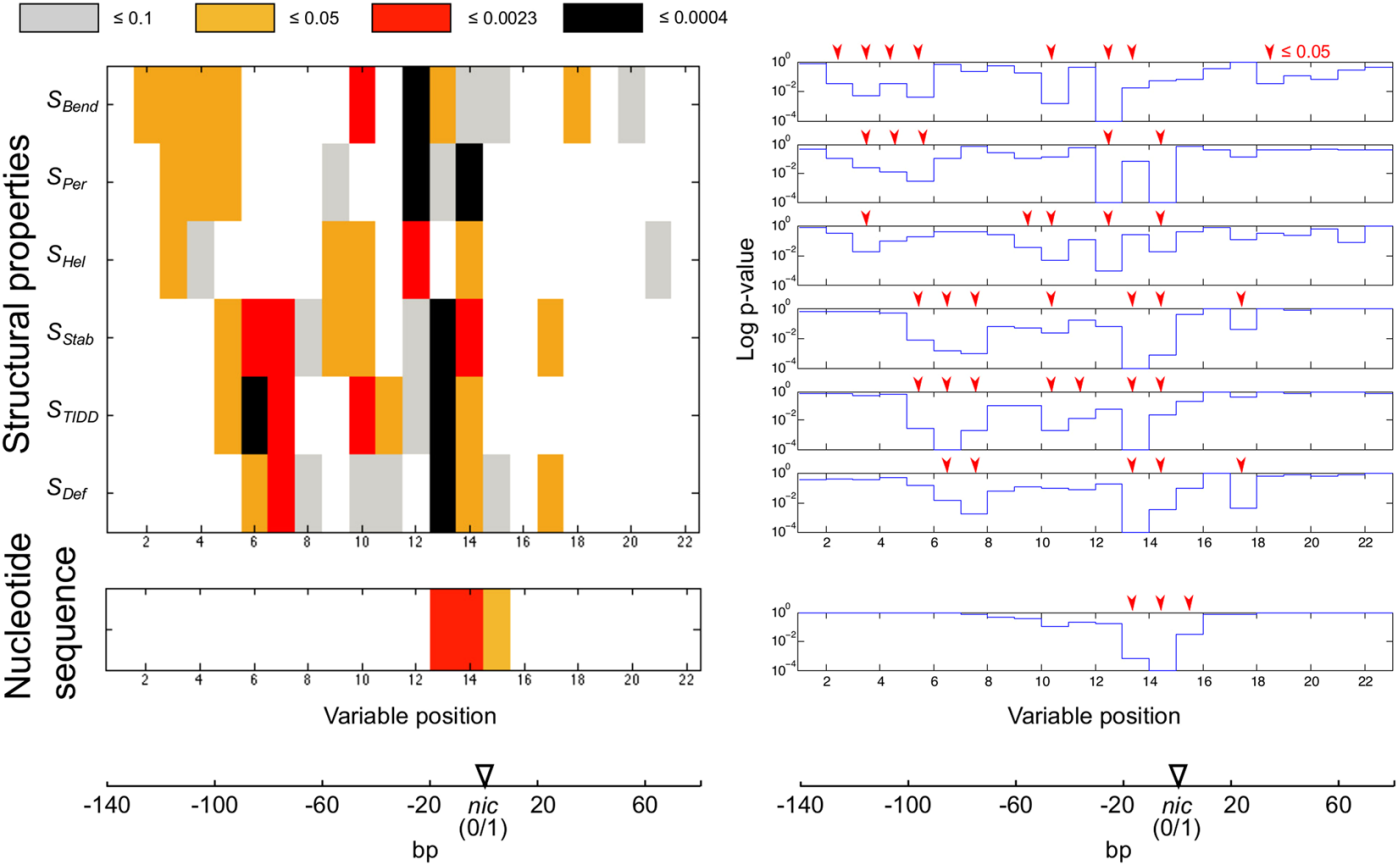
Conservation of structural variables and nucleotide sequences according to analysis of variance. Variables of 6 structural properties and nucleotide sequences in windows of 10 bp were compared at 22 positions in *oriT* regions (labeled ‘Variable position’ on the x axis). *P* values of the *F* statistic (y axis) are given at levels of significance that are (i) uncorrected (0.05) and (ii) corrected for multiple comparisons within a particular structural property or nucleotide sequence spanning 22 variables (0.0023) or (iii) whole set of 6 structural properties (0.0004).

Analysis of variance of nucleotide sequence and structural properties at the 22 variable positions in *oriT* showed that structural properties were significantly conserved at multiple *oriT* positions (Fig. 3: 1 to 2 significant positions with the most stringent corrections for multiple testing, except with property S_*Hel*_). However, nucleotide sequences were conserved only around the *nic* site (1 significant position; see Supp. Fig. S3). Up to a two fold increase of conserved positions was thus obtained with the structural variables compared to the nucleotide sequences (Fig. 3: 28% vs. 14% of positions, respectively, with uncorrected *p*).

**Figure 4:**
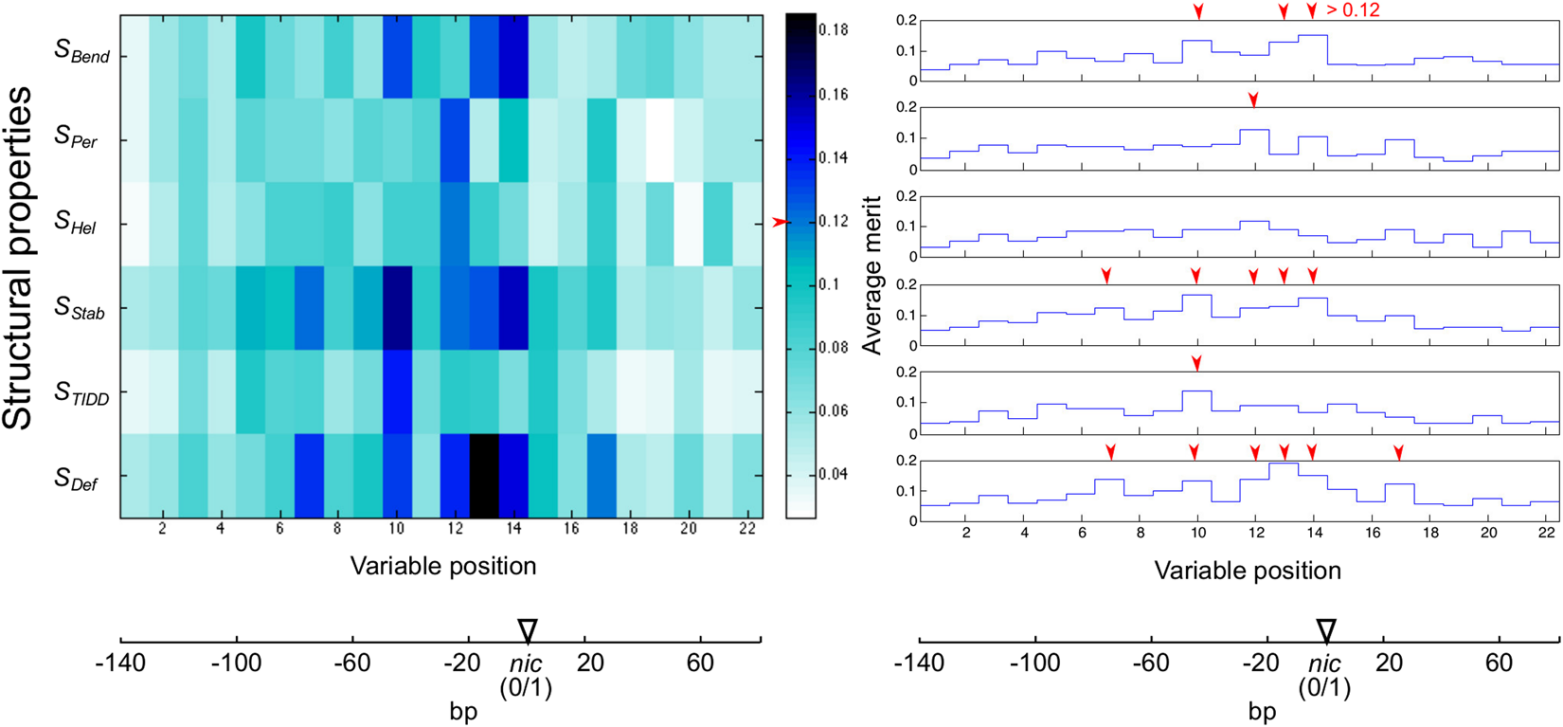
Variable analysis using the ReliefF algorithm. Relative importance (ReliefF Average merit on the y axis) of the structural variables of 6 structural properties (labeled ‘Variable position’) in the *oriT* regions is shown. The cutoff level of relative importance (Average merit) for the subset of 16 highest ranked variables and the positions of these variables are marked with red arrows.

When structural variables were ranked according to their relative importance of discrimination of MOB groups using machine learning algorithms (Supp. Table S4: ReliefF and CFS algorithms), the highest measures of classification performance were obtained with a subset of 16 highest ranked variables using the ReliefF algorithm (Fig. 4: testing models built with training dataset using testing dataset; Supp. Fig. S4 and Table S5). This was a significant improvement to using the full set of 132 variables (*p* < 0.002) as well as to the classification performance measures obtained with subsets of variables ranked according to *p*-values or the CFS algorithm (*p* < 0.006). The most informative structural properties according to the variable subset obtained with the ReliefF algorithm were DNA deformability *S*_*Def*_, duplex stability *S*_*Stab*_ and bending propensity *S*_*Bend*_ (Fig. 4: 6, 5 and 3 highest ranked variables, respectively), whereas thermally induced duplex destabilization *S*_*TIDD*_ and persistence length *S*_*Per*_ were less informative (1 variable each). No variables from helical repeats *S*_*Hel*_ were present among the highest ranked variables, though *S*_*Hel*_*12* was the 17^th^ highest ranked according to ReliefF (see Supp. Table S3).

**Figure 5:**
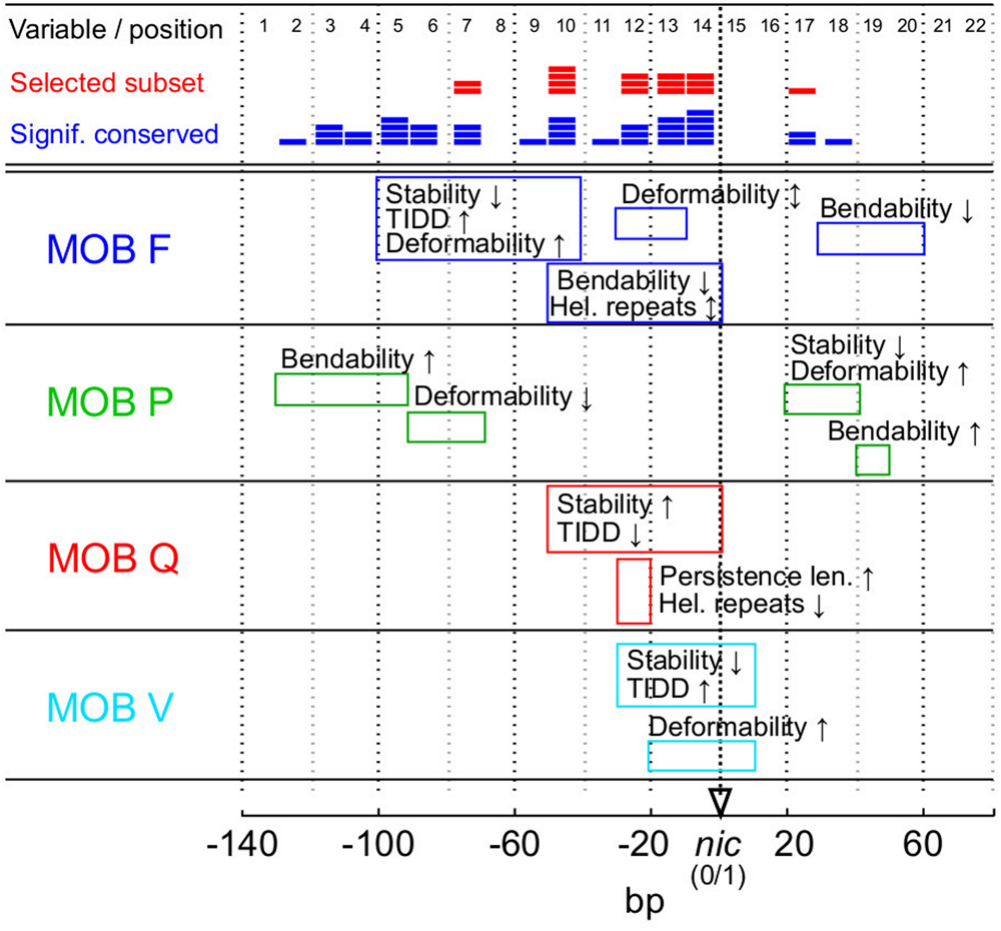
Overview of structural properties and variable analysis in *oriT* regions from four MOB groups. Shown are the most prominent structural properties that separated a particular MOB group from the other groups (see details in Supp. Fig. S5). Also depicted at specific positions are the amount of variables from the selected subset (Fig. 4, red color) and the amount of variables with significant conservation (Fig. 3, blue color).

The majority of the 16 highest ranked structural variables were upstream from *nic* (Figs 4 and 5: 15 out of 16) and over half of these (Figs 4 and 5: 9 of 16) were less than 30 bp away from *nic*. In group MOB F, in the region from −100 to −40 bp the mean stability *S*_*Stab*_*7,10*, destabilizations *S*_*TIDD*_*10* and deformability *S*_*Def*_*7,10* showed largest deviations from other groups (Supp. Fig. S5; differences were significant *p* < 0.006) and coincided with inverted repeats and auxilliary protein binding sites (Fig. 1: eg. *sbaB* and *sbyA*)^44^. Similarly, in the interval from approximately −50 to −10 bp the mean bending propensity was lower in MOB F than elsewhere (*S*_*Bend*_*10,13*, see Supp. Fig. S6; *p* < 0.001) and *S*_*Bend*_*10* coincided with an IHF binding site (Fig. 1: *ihfA*)^44,45^. In MOB P, significant increases in bending propensity *S*_*Bend*_*2–5* from −130 to −90 bp and a decrease in deformability *S*_*Def*_*6,7* from −90 to −70 bp coincided with binding site *mcb1* and inverted repeats, respectively (*p* < 0.006). The region downstream from *nic* also showed relevance for MOB P discrimination, since mean deformability *S*_*Def*_*17* and DNA stability (Supp Table S4: *S*_*Stab*_*17* is ranked just below the 16 subset) were lower and bending propensity *S*_*Bend*_*18* was higher compared to other groups (Fig. 1: positions correspond to IR5 in pC221 and TraK binding site *srk* in RP4^46^; see Supp. Fig. S7; *p* < 0.002)^46,47^. In MOB Q, mean persistence length *S*_*Per*_*12*, stability *S*_*Stab*_*12* and deformability *S*_*Def*_*12,13,14* as well as the significantly conserved amount of helical repeats *S*_*Hel*_*12* showed large deviations from other groups at around −20 bp, corresponding to locations of IRs involved in relaxase binding (Fig. 1; *p* < 0.002)^48,49^. Similarly, MOB V displayed a low mean stability *S*_*Stab*_*12,13,14* and high amount of destabilizations around −10 bp (Supp. Table S4: *S*_*TIDD*_*12,15* are ranked immediately below the 16 variable subset; all *p* < 0.001), coinciding with IRs^50,51^.

### Structure based approach enables prediction of transfer range

Using machine learning algorithms with the selected structural variables, predictive models were built that could classify input *oriT* regions into their corresponding MOB groups with high precision and recall (Supp. Table S6: *Pre*_*Test*_ = 0.975 ± 0.001, *Rec*_*Test*_ = 0.973 ± 0.001, *Pre*_*CV_200*_ = 0.958 ± 0.001, *Rec*_*CV_200*_ = 0.949 ± 0.002). Since certain elements in the training dataset were frequently inaccurately classified, we examined how their removal from the dataset affected classification performance. Results showed that removal of any elements from the training dataset negatively affected the performance of the models. Although removal of the first nine elements (see Supp. Table S6) with a classification frequency below 0.2 led to improved results of cross validations (*Pre*_*CV_64*_ increasing to 0.842 ± 0.008, *Rec*_*CV_64*_ to 0.790 ± 0.006 to *Pre*_*CV_64*_ to 0.988 ± 0.003 and *Rec*_*CV_64*_ to 0.979 ± 0.004*, P* < 0.001), testing with the 140 element dataset showed a decrease in predictive performance (*Pre*_*Test*_ = 0.975 ± 0.001, *Rec*_*Test*_ = 0.973 ± 0.001 to *Pre*_*Test*_ = 0.789 ± 0.001, *Rec*_*Test*_ = 0.763 ± 0.001, *P* < 0.001).

**Figure 6:**
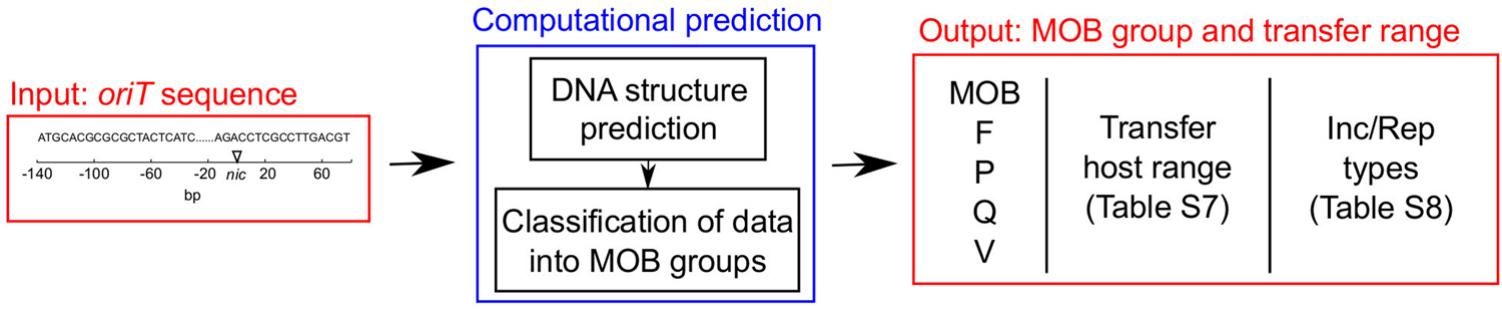
Overview of the *oriT* structure-based prediction procedure. Based on an input *oriT* sequence, the computational procedure predicts (i) the MOB group of the particular *oriT* and plasmid as well as (ii) the range of potential transfer hosts and Inc/Rep types (see Discussion). Two types of predictive classification models are available to the user, based the training sets of either 64 or 200 elements.

In order to facilitate the prediction of the plasmid transfer range using our models, we collected all currently available data into two tables^8,10,52^ (Supp. Tables S7 and S8), which link the MOB classification of plasmids with known transfer hosts and Inc/Rep types. The predictive classification models based either on the set of 64 experimentally obtained elements or the whole set of 200 elements were implemented as a webtool available at http://dnatools.eu/MOB/plasmid.html (Fig. 6). The input is a DNA sequence, which is a 230 bp long *oriT* region with the *nic* site located between positions 140 and 141. The output consists of (i) the predicted MOB group of the particular *oriT* and plasmid as well as (ii) the range of potential transfer hosts (Supp. Table S7) and Inc/Rep types (Supp. Table S8) in the MOB group, according to the data available for the training elements.

## Discussion

The approach that is currently used to classify a particular plasmid is based on analysis of amino acid sequences of relaxases and accessory proteins. Here however, we showed for the first time that plasmids can be correctly classified into MOB groups based on predicted structural properties of noncoding *oriT* sequences, without any information about the relaxase. The *oriT* regions act as relaxase recognition sites as well as enzymatic substrates for nicking. Accordingly, we can conclude that *oriT* structural properties have co-evolved with the relaxases and accessory proteins involved in the DNA recognition, nicking and transfer reactions within their particular MOB group, as theory and experimental evidence suggested^16–19^.

This is supported by the analysis of variance, which showed that within the MOB groups *oriT* regions contained significantly conserved structural properties (Fig. 3). However, the statistical procedure did not account for any possible interactions between the structural properties and structural variables, which were presumed to be important in *oriT* due to latent structural connections. We therefore performed additional analysis and selection of variables using machine learning algorithms (Fig. 4, Supp. Table S4). Ranking the variables based on their importance in discrimination of MOB groups helped us to identify the structurally informative *oriT* regions. The subset of 16 highest ranked variables (see Fig. 4, Supp. Table S4) thus included 12 variables that were determined to be significantly conserved with the analysis of variance (Fig. 3: *p* < 0.05). Of these 12 variables, 3 variables were below the corrected significance level of *p* < 0.0004 and 5 were below *p* < 0.0023 (see Fig. 3). With 3 of the 4 additional variables included in the subset of 16 highest ranked variables (Fig. 4) and not determined to be significantly conserved, *p* was below 0.1, showing a moderate degree of conservation (Fig. 3: bending propensity *S*_*Bend*_*14*, stability *S*_*Stab*_*12* and deformability *S*_*Def*_10). These variables were probably included due to variable interactions, which were also likely the reason that some of the most significant variables (4 of 7 with *p* < 0.0004) were not included in the selected subset.

The selected structural variables that enabled the most accurate classification of MOB groups were the most informative, since they coincided with experimentally determined *oriT* structural properties. By comparing the variables with *oriT* protein binding sites we observed a higher conservation of structural properties at or around specific protein binding sites than at other positions (Figs 1 and 5, Supp. Fig. S5). The region in the immediate vicinity of *nic* was the most relevant for analysis of *oriT* regions and their classification (Fig. 5: over half of the selected variables), since it is the most important for DNA relaxation. This region contains inverted repeats and well characterized binding sites in all MOB groups (Fig. 1)^11^. The structural variables around *nic* reflected specific relaxase binding and nicking properties in the particular groups of elements. For instance, formation of DNA melting bubbles and hairpins involved in relaxation separated MOB groups Q and V^50,51^ from other MOB groups (Fig. 5). As expected according to experimental data, most of the selected attributes were upstream from *nic*, since this region has a greater role in the control of relaxation than the downstream region. This was most prominent in groups MOB F and P, since they have more auxiliary protein binding sites and span farther upstream than other groups (Fig. 1)^11,53^. The downstream region also showed relevance for classification, since certain elements in MOB F and P contain downstream binding sites for auxiliary proteins (Fig. 1: RP4 and pC221 in MOB P, R388 in MOB F: deviations in mean stability *S*_*Stab*_20, deformability *S*_*Def*_20 and bendability *S*_*Bend*_20 corresponded with IR11, *p* < 0.001)^47,54,55^.

The conservation of *oriT* structural properties inside MOB groups might be a consequence of the evolutionary development of the specific relaxation systems. According to our results and the current understanding, one possible way that *oriT* regions have evolved, is that relaxases in the ancestral state were of lower specificity and targeted multiple existing *oriT*s^48,56^. These *oriT*s evolved and adapted to their particular relaxase, after which the relaxase evolved to optimize interaction and enzymatic function with the best *oriT*. In some MOB systems, this includes the acquisition of other (auxiliary) proteins to aid the process. A particular relaxase therefore defines a particular *oriT* as this enables a stable structure of genes, a low number of deletions during conjugation, stable size of plasmids as well as the optimization of levels and functioning of plasmid-coded proteins and timing of their expression^8,15,57^. However, according to the above process it is also possible that (i) certain mobile elements can carry multiple *oriT*s^58^, and (ii) *oriT* regions might be present on elements lacking relaxases to confer mobility^59,60^.

According to such *oriT* evolutionary processes as described above, we hypothesize that relaxation systems with a larger amount of auxiliary proteins, such as MOB F and P, are more mature and optimized than ones with less auxilliary proteins (e.g. MOB Q and V, see Fig. 1). They could have had a more directed or longer evolution, meaning they are evolutionarily older systems. The observations are also supported by the reported characteristics of relaxation systems and conjugative properties of the conjugative elements that carry them. In contrast to the more advanced MOB F and P systems frequently carried by conjugative and larger (>30 kb) plasmids^12^, simpler MOB Q and V systems are usually carried by mobilizable and not conjugative elements. Therefore they rely on conjugation components (see Introduction) of the host or other plasmids for transfer^12^. The elements might lack such components due to being smaller (<30 kb) and potentially less evolved, which drives them to be more promiscuous so that they can exploit horizontal gene transfer to endure negative selection pressure. This higher promiscuity relates to simplicity of the *oriT* system of MOB V, which directly possesses the structural properties required for strand separation and relaxation (Fig. 5: low stability and high amount of destabilizations near *nic*), whereas the other MOB groups require auxiliary proteins to help them achieve this^11^. Nevertheless, in plasmids from the group MOB Q both auxiliary proteins and relaxases are known to have a very low DNA-binding specificity (e.g. RSF1010)^48^ and therefore we also expect that they are more promiscuous.

The results based on conventional nucleotide sequence analysis using evolutionary distance models (p-distance and Kimura) and the low DNA sequence conservation in *oriT* regions (Fig. 3, Supp. Fig. S3) support our findings on the conservation and evolution of *oriT* structure within conjugation systems. An important restriction with the sequence based analysis was that *oriT* sequences were misaligned, resulting in large distances between sequences and the inability to determine the Kimura distance (tendency of pyrimidine or purine substitutions)^24^ for all sequences, which led to inaccurate clustering (Supp. Fig. S2). Accordingly, with regions that display a high degree of conservation of structures, such as *oriT*, a more suitable approach would be to align them based on patterns of conservation of structural properties instead of merely nucleotide sequence patterns.

The cause for low classification frequencies of certain conjugative elements (Supp. Table S6), was that most of them were independent representatives of MOB subgroups or belonged to unknown subgroups^9,10^. Comparison with classification of plasmids according to relaxase amino acid sequence conservation in Barcia *et al*.^10^ shows that in our study, the misclassified plasmids differed from other elements also according to the conservation of their cognate relaxases. In the case of plasmid pWWO from MOB subgroup F11, in Barcia *et al*.^10^ the three other plasmids in subgroup F11 were clustered together in the same branch based on relaxase classification (bootstrap confidence of 99%), while pWWO was in a separate branch (bootstrap confidence of 99%). Similarly, the plasmid pAB6 from MOB Q1 was clustered separately from the other elements (bootstrap confidence of 100%). In the case of plasmids pTA1060 (MOB subgroup V1) and pIP421 (MOB V4), no possible cause for misclassification could currently be determined, since the phylogeny of all elements of MOB V is currently unavailable^10^. The results indicate that the phylogeny of *oriT* subtrates reflects that of their cognate relaxases (initial tests of classification using the whole dataset and MOB subgroups resulted in over 88% accuracy of cross-validations).

Since researchers require fast procedures to identify a plasmids MOB group and transfer range, we implemented the *oriT* structure-based procedure as a webtool (see Fig. 6). Although based on mere MOB classification we cannot predict the exact receiving host of a plasmid, we can restrict the selection to a range of hosts, where such types of plasmid have been found previosly. Given that the potential host range of a plasmid is not defined only by plasmid transfer, but also by the propensity of the plasmid to stabilize in the subsequent generations of the bacterial host^8,10,52^, two separate ranges can be distinguished (see Fig. 6): (i) the range of potential transfer hosts, based on the hosts of plasmids used for training the models (Supp. Table S7), and (ii) the range of potential incompatibility and replication (Inc/Rep) types that can help determine the replication host range (Supp. Table S8). Since they define entire transfer systems, MOB groups are one of the factors by which to determine the transfer host range, which is generally wider than the replication host range^10,12^. In *Gammaproteobacteria*, the plasmid replication (Rep) types were shown to be much more restrictive (in the plasmids they can amplify) than the MOB types^8^. However, since MOB groups were shown to include highly conserved distributions of Inc/Rep types^8,52^ and to describe complete plasmid backbones^12,15^, they can potentially provide important information on plasmid stability and behaviour in the host. Moreover, studies have shown that plasmid transfer host ranges can also be defined by other components of the conjugation system, such as the T4SS (mating complex) proteins^12,52^, which will undoubtedly serve as the basis for future improvements.

The significance of our results is that the transfer range of an AMR carrying plasmid can be determined merely by analysis of the structure of the *oriT* sequence instead of whole relaxase genes. Since they can facilitate binding of relaxases even in trans^48,59,60^, *oriT* substrates are the most elementary prerequsites for DNA mobility. Considering that there are potentially more *oriT* regions than relaxase genes^58–60^, as well as the algorithmic differences between nucleotide and protein sequence analysis, we presume that the identification and characterization of *oriT* substrates can potentially greatly improve the accuracy of predictions of plasmid mobility and hosts, over protein-based analyses. Consequently, the present method facilitates development of novel solutions to decrease AMR incidence with antibiotic treatments, since for a given AMR carrying plasmid the potential routes of transfer within its MOB group can guide the optimization of antibiotic treatments that limit the growth of the most frequent hosts.

## Electronic supplementary material

Supplementary Information
