# Supplementary material for: DNA structure at the plasmid origin-of-transfer indicates its potential transfer range
Source: Sci Rep. 2018 Jan 29;8:1820. doi: 10.1038/s41598-018-20157-y (PMC5789077; doi:10.1038/s41598-018-20157-y)
Supplement: Supplementary file 1 — Supplementary Information [file 41598_2018_20157_MOESM1_ESM.pdf]

DNA structure at the plasmid origin-of-transfer indicates its potential transfer range

Jan Zrimec[1,2,*] & Aleš Lapanje[1,3,4,**]

[1] Institute of Metagenomics and Microbial Technologies, Clevelandska 19, 1000 Ljubljana, Slovenia

[2] Faculty of Health Sciences, University of Primorska, Polje 42, 6320 Izola, Slovenia

[3] Saratov State University, Astrakhanskaya 83, Saratov, Russian Federation

[4] Institute Jozef Stefan, Department of Environmental Sciences, Jamova 39, 1000 Ljubljana, Slovenia

* Tel.: +386 41 925 729; E-mail address: janzrimec@gmail.com

** Tel.: +386 41 253 526; E-mail address: lapanje.ales@gmail.com

Supplementary information:

Supplementary methods S1 – S3

Supplementary figures S1 – S7

Supplementary tables S1 – S8

References

Supplementary methods S1

Tests of normality and equality of variance were rejected at the 0.05 significance level for approximately 70% of the structural variables (Bartlett's test and other normality tests). Box's M test for multivariate normality and equality of covariance could not be performed due to insufficient size of the dataset.

Supplementary methods S2

The total sum of squares was given by

$$SS_{Total} = \frac{1}{N} \sum_{i=1}^{N-1} \sum_{j=i+1}^{N} d_{ij}^2 \quad , (1)$$

where $N = an$ was the total number of observations, a the number of groups, n the number of observations in each group, and d_{ij} was the distance between observation i = 1,...,N and observation j = 1,...,N [33]. The within group residual sum of squares was

$$SS_{Within} = \sum_{i=1}^{N-1} \sum_{j=i+1}^{N} \epsilon_{ij} \frac{d_{ij}^2}{n_k} \quad , (2)$$

where ε_{ij} was equal to 1 if the observation i and observation j were in the same group, otherwise it equaled zero, and n_k was the number of observations in the k-th group. Accordingly, the between group sum of squares was

$$SS_{Between} = SS_{Total} - SS_{Within} \quad (3)$$

and a pseudo F statistic to test the multivariate hypothesis of equivalence of means was constructed according to

$$F = \frac{SS_{Between} / (a-1)}{SS_{Within} / (N-k)} \quad . (4)$$

The distribution of F under the null hypothesis of no differences among group means was evaluated by performing 1000 bootstrap repetitions, since this procedure resulted in more rigorous statistics and was more accurate than permutations. With individual structural and sequence variables, 3000 bootstrap repetitions were performed to correct for multiple comparisons. P values were calculated according to

$$P = \frac{(No.of\ F_{Bootstrap} \geq F)}{(Total\ no.of\ F_{Bootstrap})} \quad . (5)$$

To compare the different data representations, *F* was scaled to the interval [0,1] based on the bootstrap distributions defining the maximum (1) and minimum values (0)

$$F_{Scaled} = \frac{F - min(F_{Bootstrap})}{max(F_{Bootstrap}) - min(F_{Bootstrap})} \quad . (6)$$

The hypothesis that two *F* statistics differed significantly was tested by obtaining the distribution of $F_1 - F_2 > 0$ from bootstrap replicates.

Supplementary methods S3

The classification tests were evaluated with the following classification measures:

(i) Average Classification Accuracy (*ACA*), which is the ratio of the number of correctly predicted elements to number of all predictions made,

(ii) Precision (*Pre*), which is the fraction of predictions that are known to be true (positive predictive value of the classifier),

(iii) Recall (*Rec*), which is the fraction of known classes that were successfully predicted (true positive rate),

(iv) Cohen's Kappa statistic (*CK*), which is a measure of the difference between observed agreement of predictions and the expected agreement according to chance alone [38],

(v) Matthew correlation coefficient (*MCC*), which is a balanced measure of true and false predictions [39],

(vi) Area under ROC curve (AUC), which is a measure of the discriminability of classes showing the probability of correct predictions [40,41].

Supplementary figures

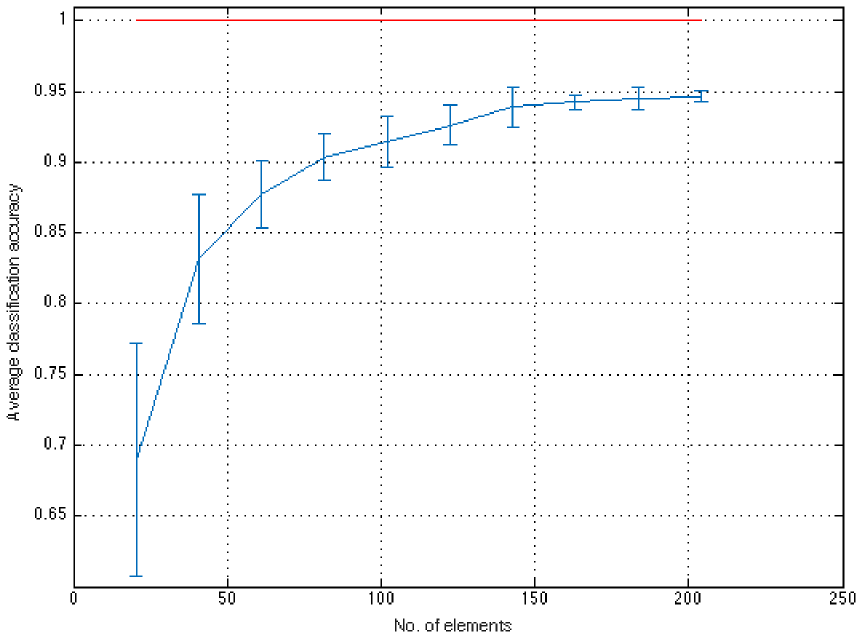

Figure S1. Learning curves. Learning curves of training (red – all 100%) and testing with 10-fold cross validations (blue) on dilutions of 204 element dataset (10 repetitions per run, 95% confidence bounds shown).

Supplementary_figure_S5A.png
Supplementary_figure_S5B.png
Supplementary_figure_S5C.png
Supplementary_figure_S5D.png
Supplementary_figure_S5E.png
Supplementary_figure_S5F.png
Supplementary_figure_S5G.png
Supplementary_figure_S5H.png

Figure S2. Dendrograms obtained by clustering of elements. Clustering was performed with aligned (A-D) and unaligned *oriT* sequences (E-H), based on the p-distance sequence similarity measure and the neighbor joining method (A,B,E,F) and Kimura two-parameter sequence similarity measure and the maximum likelihood method (C,D,G,H). Trees were condensed using a bootstrap confidence treshold value of (A,C,E,G) 80% and (B,D,F,H) 50%. Estimated average classification accuracies were (A) 0.098 ± 0.118, (B) 0.110 ± 0.104, (C) 0.060 ± 0.032, (D) 0.097 ± 0.067, (E) 0.063 ± 0.031, (F) 0.082 ± 0.045 (G) 0.039 ± 0.011 and (H) 0.051 ± 0.024 (95% confidence bounds shown).

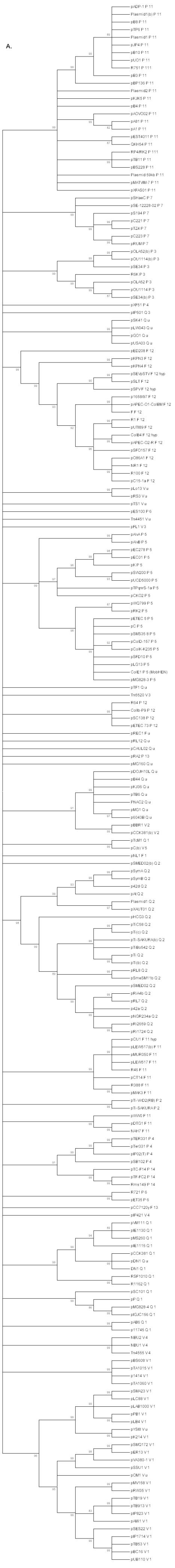

B.

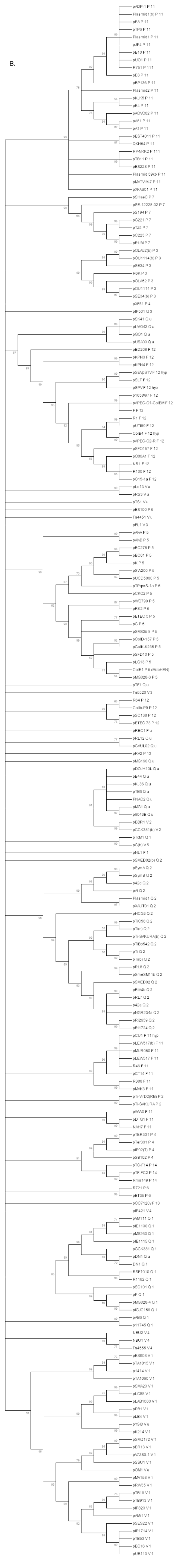

C.

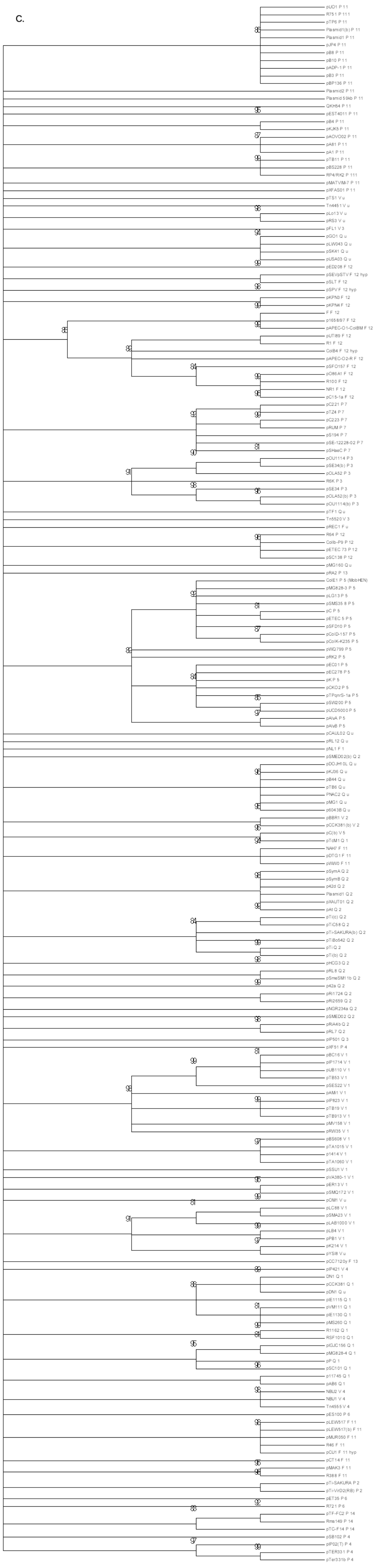

D.

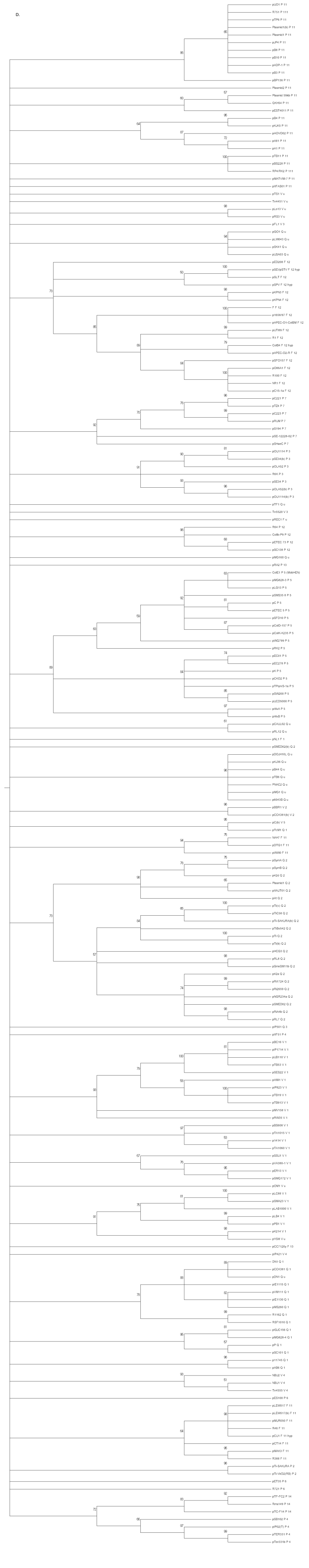

E.

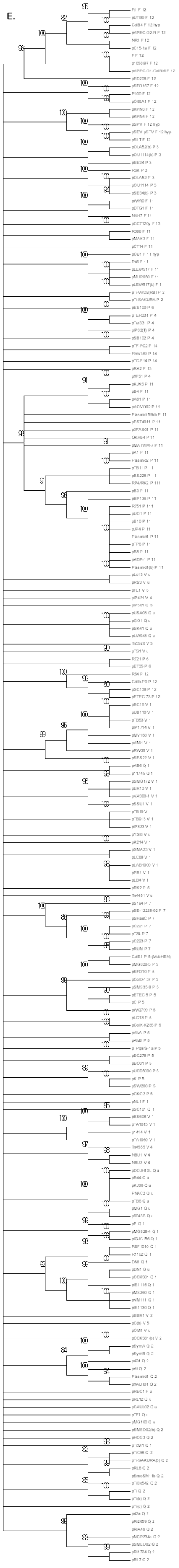

F.

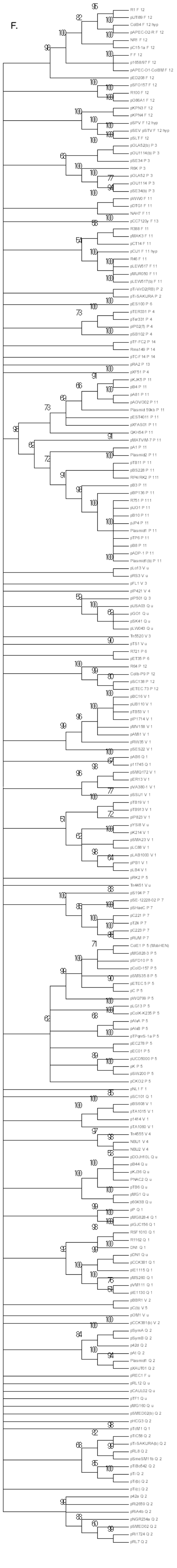

G.

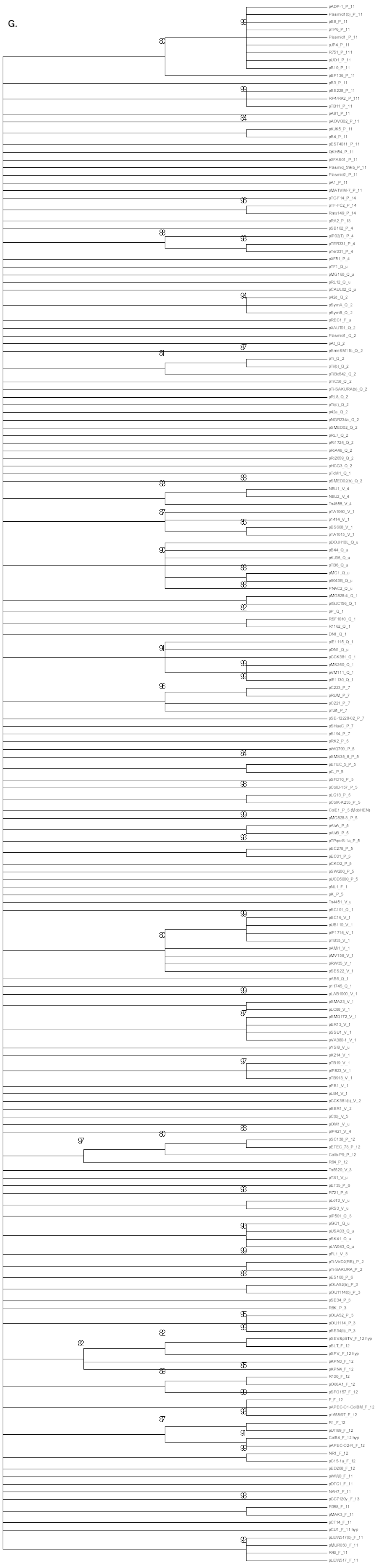

H.

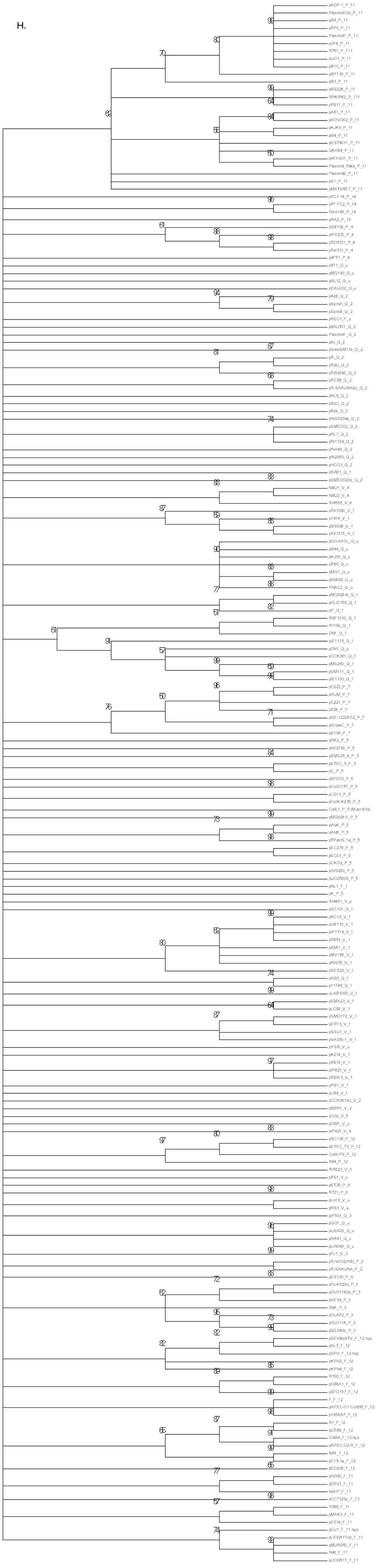

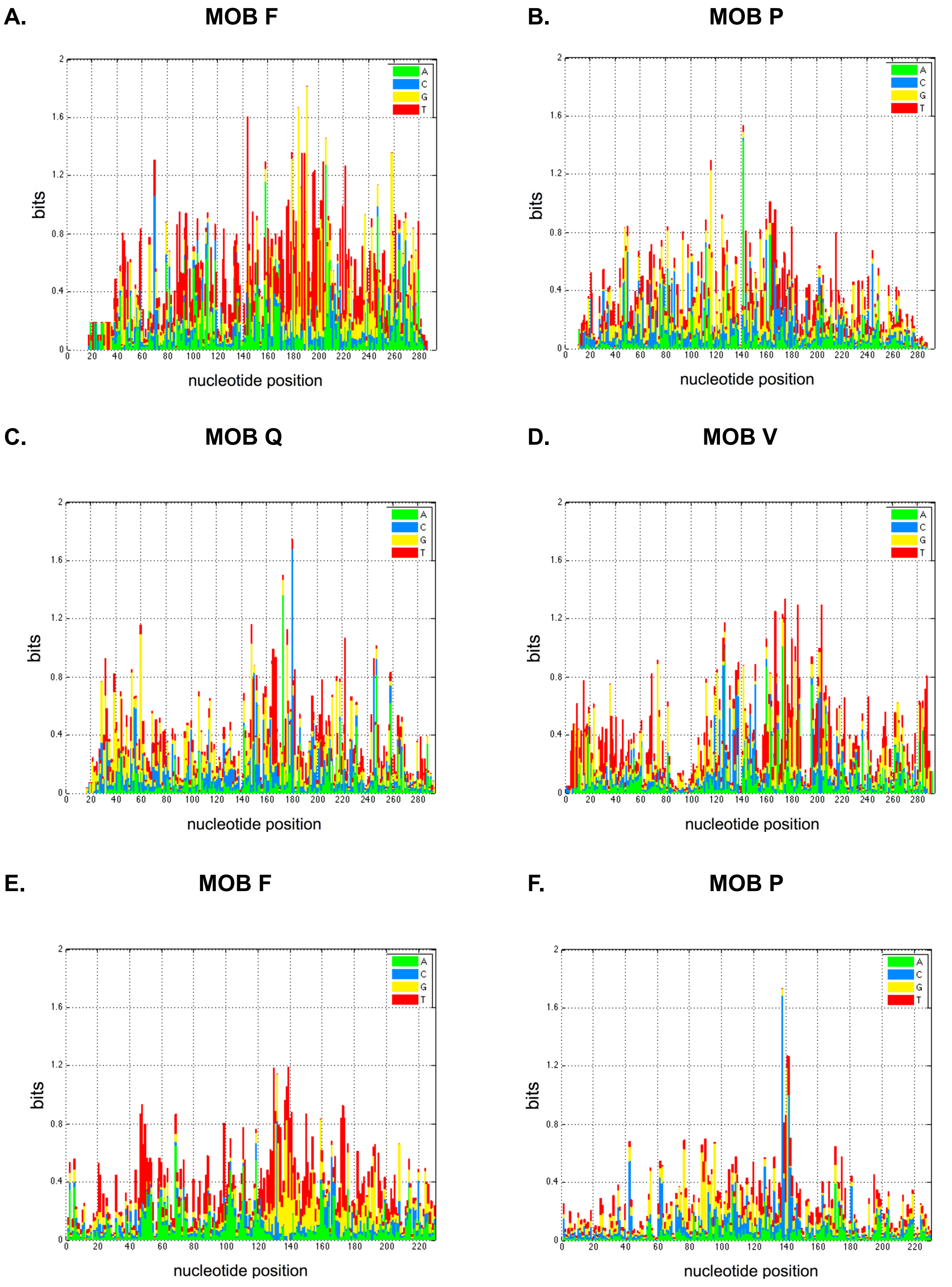
A.
MOB F
B.
MOB P
C.
MOB Q
D.
MOB V
E.
MOB F
F.
MOB P
A
C
G
T
bits
nucleotide position

G. **MOB Q**

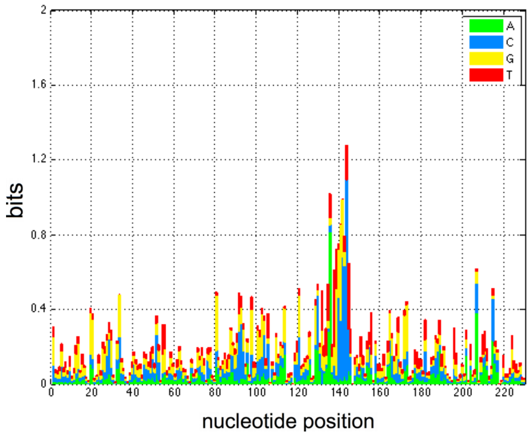

H. **MOB V**

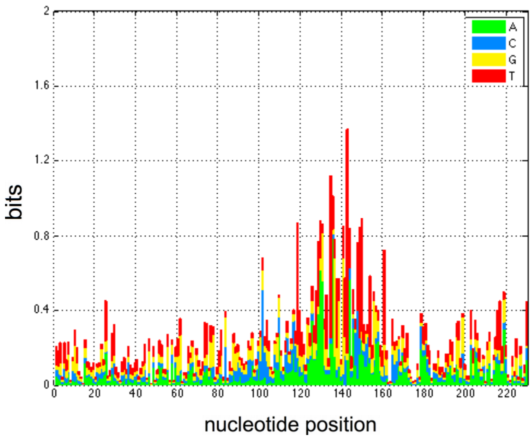

Figure S3. Sequence conservation of *oriT* regions within each MOB group. The information content per nucleotide in *oriTs* of MOB F (32 elements), P (77 el.), Q (55 el.) and V (40 el.), is shown for aligned (A-D) and unaligned sequences (E-H). The overall information content for all 200 elements was low, as the average information content per nucleotide was 0.138 ± 0.014 bits and 0.074 ± 0.010 bits (95% confidence bounds given) with aligned and unaligned *oriT*s, respectively. This indicated low sequence conservation of *oriTs*, whereas *oriT* regions of individual MOB groups showed slightly greater sequence conservation. Average information content was (A) 0.477 ± 0.041 bits, (B) 0.316 ± 0.028 bits, (C) 0.323 ± 0.031 bits, (D) 0.352 ± 0.032 bits, (E) 0.360 ± 0.032 bits, (F) 0.238 ± 0.029 bits, (G) 0.197 ± 0.024 bits and (H) 0.244 ± 0.028 bits.

A.

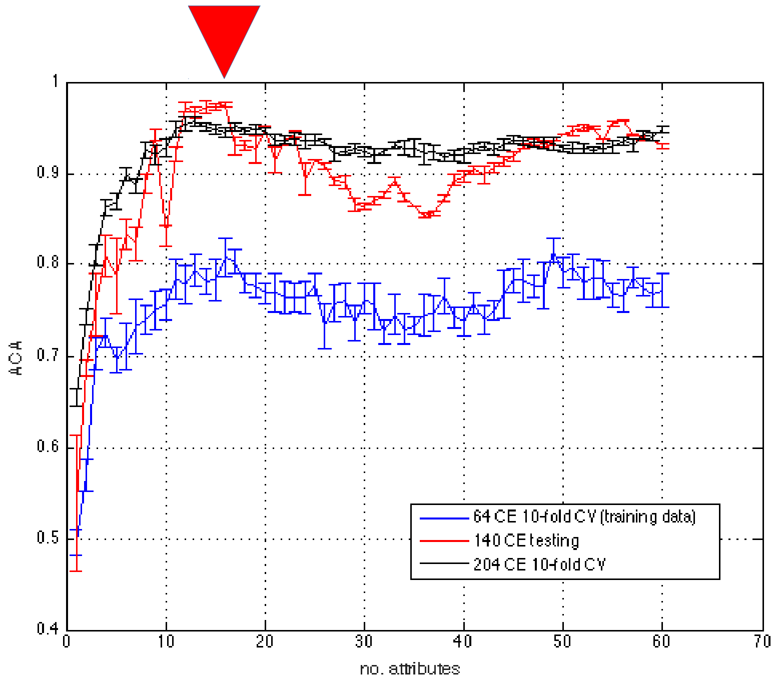
ACA
no. attributes
64 CE 10-fold CV (training data)
140 CE testing
204 CE 10-fold CV

B.

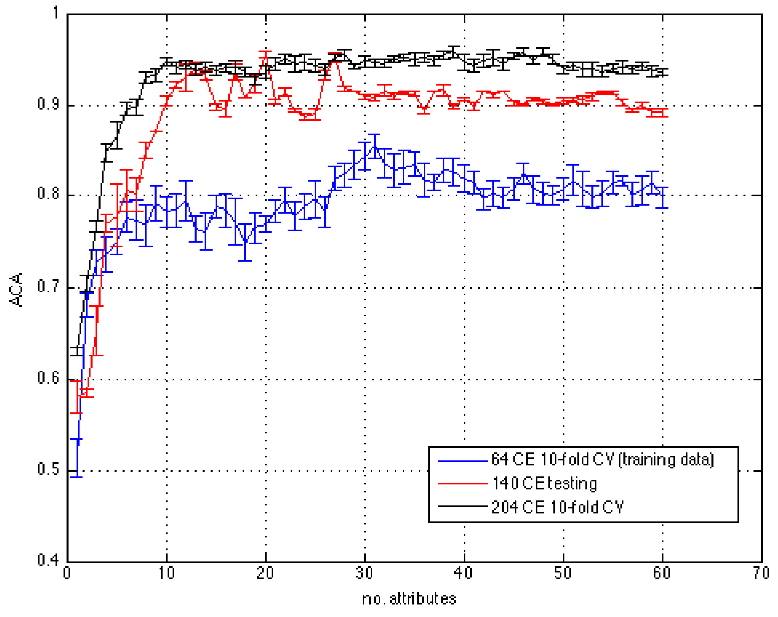
ACA
no. attributes
64 CE 10-fold CV (training data)
140 CE testing
204 CE 10-fold CV

C.

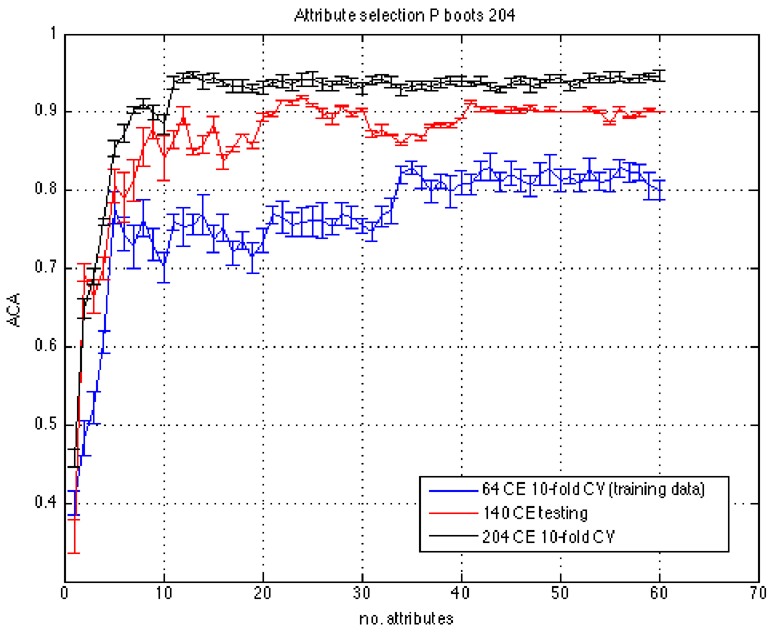

Figure S4. Evaluation of ranked variables with a backward variable selection procedure. Variables were ranked using algorithms ReliefF (A) and Csf (B) as well as based on *P* values of analysis of variance. Ranked variables were then evaluated with backward variable selection. Shown is the average classification accuracy of 10 repetitions of classification tests. Bars depict 95% confidence bounds. The best subset of 16 variables according to testing with the set of 136 elements is marked with a red arrow in (A).

Supplementary_figure_S5.png

Figure S5. Predicted DNA structural properties in *oriT* regions from four MOB groups. Shown are mean structural variables according to MOB groups F (blue), P (green), Q (red) and V (cyan) in the set of 200 elements. The subset of 16 highest ranked variables obtained with variable selection is numbered and marked with red circles on the secondary x-axis as well as the following 16 highest ranked variables with green circles (see Supplementary table S4). Significantly conserved positions are specified with blue boxes.

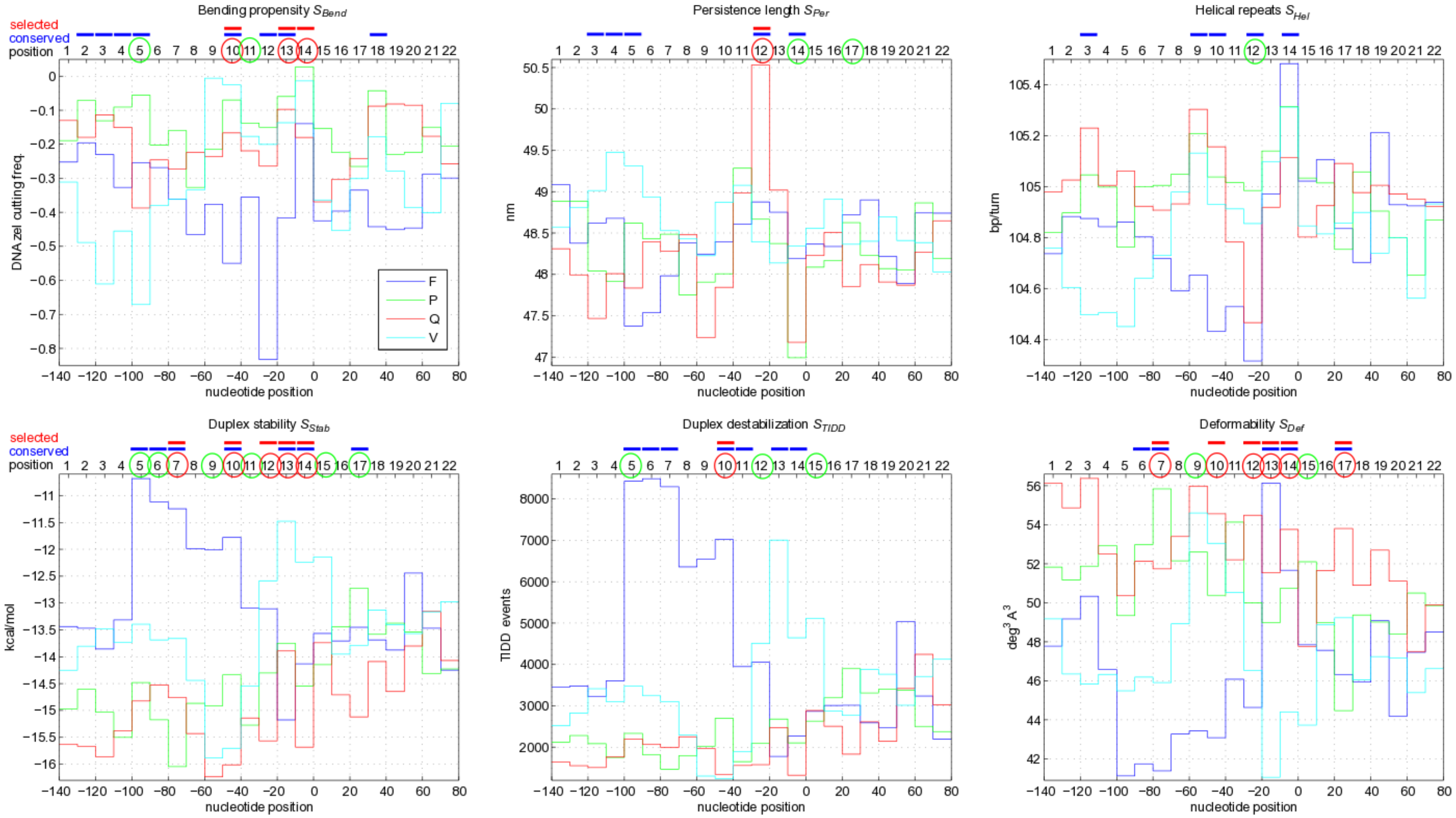
Bending propensity S_{Bend}
Persistence length S_{Per}
Helical repeats S_{Hel}
Duplex stability S_{Stab}
Duplex destabilization S_{TIDD}
Deformability S_{Def}
selected
conserved
position
DNA zel cutting freq.
nm
bp/turn
kcal/mol
TIDD events
deg^3 A^3
nucleotide position
F
P
Q
V

A.

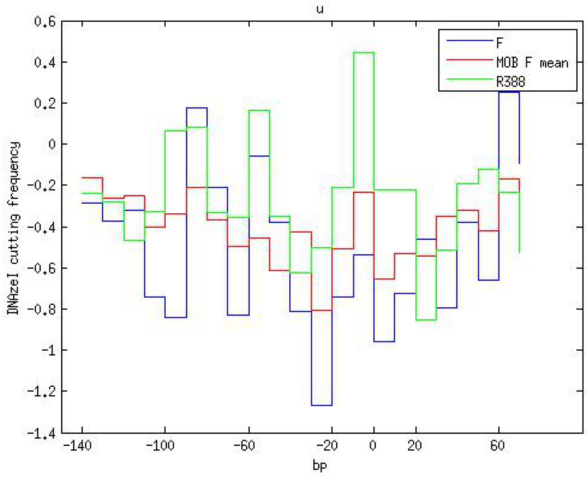

B.

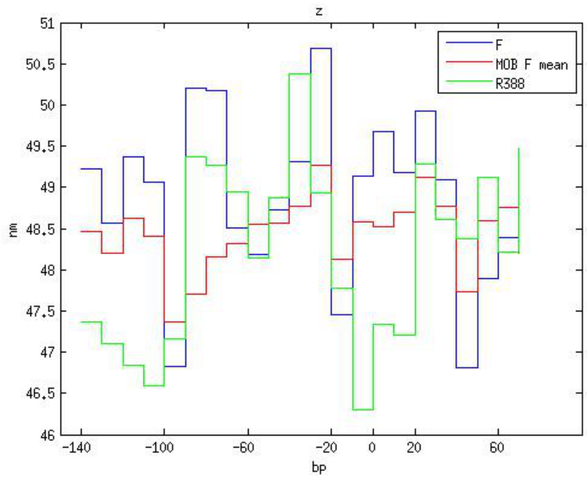

Figure S6. Bending propensity S_{Bend} and persistence length S_{Per} of MOB F plasmids. Analysis of single plasmids was performed, since the mean bending propensity in MOB F was low despite IHF binding sites in certain MOB F plasmids indicating highly curved regions (e.g. F, R388) [34,35]. (A) bending propensity S_{Bend} and (B) persistence length S_{Per} of plasmids F, R388 and mean over all elements in MOB F. Predicted bending propensity S_{Bend} according to DNAzeI cutting frequency shows similar profiles of peaks in F and R388 according to location but differing in amplitude, which correspond to the nicking and protein binding sites (peaks at -20 and 0 bp and -60: IHF binding sites, Fig. 1: *ihfA*; peaks at approx -90 bp: *sbaB* and *sbyA*). This is similar with the peaks in persistence length S_{Per} at -40 to -20bp and approx. -80 bp.

A.

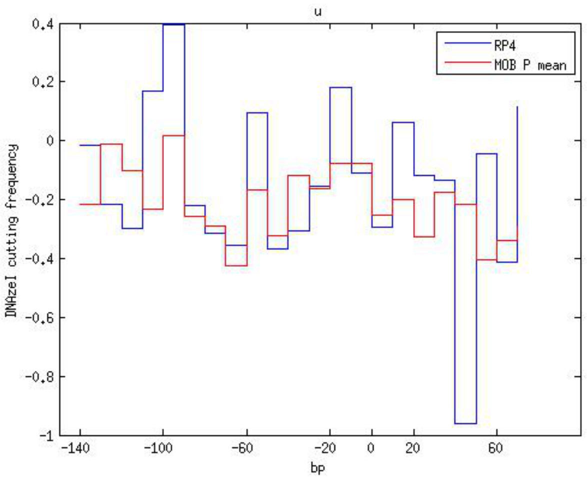

B.

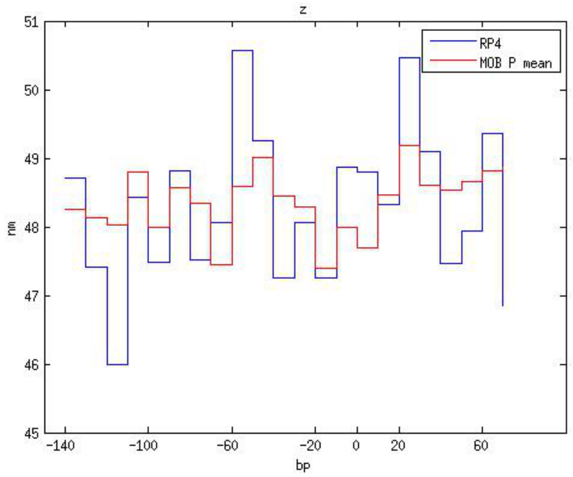

Figure S7. Bending propensity S_{Bend} and persistence length S_{Per} of plasmid RP4 in MOB P. We analysed if the bending propensity corresponded to intrinsic bends related to the binding site *srk* in plasmid RP4 [40]. (A) bending propensity S_{Bend} and (B) persistence length S_{Per} of plasmid RP4 and mean over all elements in MOB P. In the upstream region RP4 displays deviations from the mean at multiple concurrent locations in S_{Bend} and S_{Per}: approx. 20 bp, 40 bp and 60 bp, which might correspond with the *srk* binding sites (Fig. 1).

Supplementary tables

Table S1. Conjugative elements in four mobility (MOB) groups used in the study. Elements from MOB F, P, Q and V were used to study the conservation of DNA structural properties in *oriT* regions. The following parameters are additionally specified: subgroups (hypothetical are marked with 'hyp'), relaxase enzyme nicking sites *nic* (in the middle of the nucleotide sequences), accession numbers of the elements in the Genbank database, references in the literature for *nic* sites and *oriT* regions.

Element	MOB	Subgroup	*nic* (5'-N5/N5-3')	Genbank	Reference
pNL1	F	1	CGCTGACACC	NC_002033	(Guasch et al., 2003)
R388	F	11	GCTATAGACA	BR000038	(Llosa et al., 1995)
R46	F	11	GCTATACACC	AY046276	(Coupland et al., 1987)
pCT14	F	11	GCTATACACC	DQ126685	(Bramucci et al., 2006; Paterson and Iyer, 1997)
pWW0	F	11	TCAATACACA	NC_003350	(Greated et al., 2002)
pCU1	F	11 hyp	GCTATACACC	M81668	(Paterson and Iyer, 1997)
F	F	12	GCACCACACC	NC_002483	(Matson and Morton, 1991; Reygers et al., 1991)
R1	F	12	GCACCACACC	X00783	(Sut et al., 2009)
R100	F	12	CACCACACAC	NC_002134	(Abo and Ohtsubo, 1995)
pED208	F	12	GTCCCACACC	AF411480	(Laurenzio et al., 1991)
PSEV / pSTV	F	12 hyp	AGCACCACAC	AF389528	(Chu et al., 2002)
pSPV	F	12 hyp	AGCACCACAC	AF389529	(Chu et al., 2002)
ColB4	F	12 hyp	GCACCACACC	M15134	(Finlay et al., 1986)
pCC7120y	F	13	TTTTCGCACA	NC_003267	(Guasch et al., 2003)
RP4 / RK2	P	11	CCGGGCAGGA	X54459	(Pansegrau et al., 1988)
R751	P	11	GCGGGCAGGA	U67194	(Pansegrau et al., 1988)
R64	P	12	CGGGACAGGA	NC_005014	(Furuya et al., 1991)
pRA2	P	13	GGGGACAGGG	NC_005909	(Rawlings and Tietze, 2001)
pTF-FC2	P	14	CAATACAGGA	M57717	(Rohrer and Rawlings, 1992)
pTC-F14	P	14	TTGAACAGGA	NC_004734	(Francia et al., 2004)
pTi - VirD2 (RB)	P	2	TTTGACAGGA	AJ237588	(Tzfira et al., 2004)
R6K	P	3	CGATGCAGGA	X05644	(Avila et al., 1996)
pIP02(T)	P	4	TATTGCAGGA	NC_003213	(Tauch et al., 2002)
pTER331	P	4	TATTGCAGGA	NC_010332	(Mela et al., 2008)
pXF51	P	4	TAGGGCAGGA	NC_002490	(Tauch et al., 2002)
pSB102	P	4	TATTGCAGGA	NC_003122	(Tauch et al., 2002)
ColE1	P	5	TTAAGCCAGT	J01566	(Varsaki et al., 2009)
R721	P	6	ACAGGCACGA	AP002527	(Dunn et al., 2005)
pES100	P	6	ATAGGCACGA	NC_006842	(Dunn et al., 2005)
pC221	P	7	TTTGGCAAGC	NC_002129	(Caryl et al., 2004)
RSF1010	Q	1	GAGGGCGCAC	NC_001740	(Scherzinger et al., 1992)
R1162	Q	1	GAGGGCGCAT	M13380	(Bhattacharjee and Meyer, 1991)
pSC101	Q	1	AGGGCGCACT	NC_002056	(Francia et al., 2004)
pP	Q	1	GGATGCGCAC	NC_003455	(Francia et al., 2004)
pAB6	Q	1	TAATGCCCAC	AF126482	(Francia et al., 2004)
pTiC58	Q	2	AGGGCGCAAT	AF010180	(Cook and Farrand, 1992)
p42a	Q	2	AAGGGCGCAA	NC_007762	(Tun-Garrido et al., 2003)
pSymA	Q	2	GAGGGCGCAA	NC_003037	(Pérez-Mendoza et al., 2006)
pSymB	Q	2	GAGGGCGCAA	NC_003078	(Pérez-Mendoza et al., 2006)
pNGR234a	Q	2	AAGGGCGCAA	NC_000914	(Pérez-Mendoza et al., 2006)
Plasmid 1	Q	2	GAGGGCGCAA	NC_008242	(Pérez-Mendoza et al., 2006)
pIP501	Q	3	AAGGGCGCAC	L39769	(Wang and Macrina, 1995)
pGO1	Q	u	GTAAGGGCGC	U50629	(Climo et al., 1996)
pTF1	Q	u	AGGGCGCACT	X52699	(Drolet et al., 1990)
pMG160	Q	u	ATGGCGCACA	NC_004527	(Francia et al., 2004)
pDN1	Q	u	AGGGCGCACT	NC_002636	(Rawlings and Tietze, 2001)
pKJ36	Q	u	TAGGGCGCAC	NC_002635	(Francia et al., 2004)
pMV158	V	1	ATAACACACT	NC_010096	(Farías and Espinosa, 2000)
pLAB1000	V	1	TATAACCCAC	M55222	(Smith and Parker, 1998)
pTA1060	V	1	TGTAACGCAC	NC_001766	(Smith and Parker, 1998)
pVA380-1	V	1	TGTAACGTAC	L23803	(Smith and Parker, 1998)
pIP823	V	1	TATAGCACAC	U40997	(Smith and Parker, 1998)
pIP1714	V	1	ATAACACACT	AF015628	(Francia et al., 2004)
pBBR1	V	2	GTATACTCAC	X66730	(Szpirer et al., 2001)
pFL1	V	3	TCTAGTGTTC	NC_002132	(Francia et al., 2004)
Tn5520	V	3	CGTAGCTTAT	AF038866	(Vedantam et al., 2006)
pIP421	V	4	TATAGCACAC	Y10480	(Vedantam et al., 2006)
NBU1	V	4	TATAGCCCAC	NC_006373	(Vedantam et al., 2006)
Tn4555	V	4	TATAGCCCAC	U75371	(Vedantam et al., 2006)
NBU2	V	4	TATAGCCCAC	AF251288	(Wang et al., 2000)
pLo13	V	u	ACTAACTTGC	M95954	(Vedantam et al., 2006)
pTS1	V	u	GTGTACTTAC	NC_002650	(Francia et al., 2004)
pOM1	V	u	TCTAACTCAC	L31579	(Francia et al., 2004)
Tn4451	V	u	TGTAACCCAC	U15027	(Vedantam et al., 2006)

Table S2. Conjugative elements in four mobility (MOB) groups used in the study – testing dataset.
The following parameters are specified: subgroups, relaxase enzyme nicking sites *nic* (in the middle of the nucleotide sequences), accession numbers of the elements in the Genbank database, position of *oriT* in the sequence.

Element	MOB	Subgroup	*nic* (5'-N5/N5-3')	Genbank	Position
pMAK3	F	11	GCTATAGACA	NC_009982	16180
pLEW517	F	11	GCTATACACC	NC_009132	61233
pMUR050	F	11	GCTATACACC	NC_007682	46883
pAPEC-O2-R	F	12	GCACCACACC	NC_006671	39750
pREC1	F	u	GGTGCGAAGG	NC_007486	45160
pLEW517	F	11	GCTATACACC	NC_009132	3131
pDTG1	F	11	TCAATACACA	NC_004999	64075
NAH7	F	11	TCAATACACA	NC_007926	19974
NR1	F	12	GCACCACACA	NC_009133	50565
pC15-1a	F	12	GCACCACACA	NC_005327	50514
pO86A1	F	12	CACCACACAC	NC_008460	64944
p1658/97	F	12	GCACCACACC	NC_004998	2141
pSFO157	F	12	CACCACACCC	NC_009602	88570
pSLT	F	12	AGCACCACAC	NC_003277	60689
pUTI89	F	12	GCACCACACA	NC_007941	77483
pAPEC-O1-ColBM	F	12	GCACCACACC	NC_009837	18133
pKPN3	F	12	ATGACCACAC	NC_009649	140428
pKPN4	F	12	ATCACCACAC	NC_009650	72313
pTB11	P	11	CCGGGCAGGA	NC_006352	55494
pBS228	P	11	CCGGGCAGGA	NC_008357	80287
pKJK5	P	11	GCGGGCAGGA	NC_008272	44910
pB3	P	11	GCGGGCAGGA	NC_006388	45893
pA81	P	11	GCGGGCAGGA	NC_006830	68723
pA1	P	11	GCGGGCAGGA	NC_007353	16808
pB4	P	11	GCGGGCAGGA	NC_003430	72110
pUO1	P	11	GCGGGCAGGA	NC_005088	56631
pB10	P	11	GCGGGCAGGA	NC_004840	31393
pJP4	P	11	GCGGGCAGGA	NC_005912	85849
Plasmid 1	P	11	GCGGGCAGGA	NC_007337	85849
pTP6	P	11	GCGGGCAGGA	NC_007680	52506
pB8	P	11	GCGGGCAGGA	NC_007502	46176
Plasmid 2	P	11	GCGGGCAGGA	NC_006824	58532
pMATVIM-7	P	11	GAGGGCAGGA	NC_009739	22312
pEST4011	P	11	GCGGGCAGGA	NC_005793	74414
pBP136	P	11	GCGGGCAAGA	NC_008459	39464
pXFAS01	P	11	CGGGGCAGGA	NC_010579	26923
QKH54	P	11	CCGGGCAAGA	NC_008055	68272
Plasmid_59kb	P	11	CAGGGCAAGA	NC_009704	50562
pSC138	P	12	CGGGACAGGA	NC_006856	81174
pETEC_73	P	12	CGGGACAGGA	NC_009788	58887
Rms149	P	14	CTTAACAGGA	NC_007100	33045
pC223	P	7	TTTGGCAAGC	NC_005243	1869
pSE-12228-02	P	7	TTTGGCAAGC	NC_005007	3103
pRUM	P	7	TTTGGCAAGC	NC_005000	18333
pSHaeC	P	7	TTTGGCAAGC	NC_007171	5814
pS194	P	7	TTTGGCAAGC	NC_005564	951
pET35	P	6	CCGAGGCACG	NC_010696	26846
pTZ4	P	7	TTTGGCAAGC	NC_010111	3150
pOLA52	P	3	GCAGGATAGG	NC_010378	30784
pOU1114	P	3	GCAGGATAGG	NC_010421	23882
pETEC_5	P	5	CTAAGCCAGT	NC_009791	218
pSMS35_8	P	5	CTAAGCCAGT	NC_010485	2928
pTPqnrS-1a	P	5	TTAAGCCAGT	NC_009807	3493
pAlvA	P	5	TTAAGCCAGT	NC_005910	4093
pAlvB	P	5	TTAAGCCAGT	NC_005911	4185
pAOVO02	P	11	GCGGGCAGGA	NC_008766	55380
pADP-1	P	11	GCGGGCAGGA	NC_004956	11997
Plasmid 1	P	11	GCGGGCAGGA	NC_007337	14719
CoIIb-P9	P	12	CGGGACAGGA	NC_002122	39635
pTi-SAKURA	P	2	TTTGACAGGA	NC_002147	197028
pTer331	P	4	TATTGCAGGA	NC_010332	10787
pSE34	P	3	GCAGGATAGG	EU219533	3483
pC	P	5	TTAAGCCAGT	NC_003457	774
pLG13	P	5	TTAAGCCAGT	NC_005019	860
pWQ799	P	5	ATAAGCCAGT	L39794	921
pMG828-3	P	5	TTAAGCCAGT	NC_008488	642
pSFD10	P	5	TTAAGCCAGT	NC_003079	1017
pRK2	P	5	TGCCAACATA	NC_005970	1158
pColD-157	P	5	TTAAGCCAGT	Y10412	3494
pColK-K235	P	5	TTAAGCCAGT	NC_006881	5477
pK	P	5	GTAAGCCAGT	NC_003456	719
pCKO2	P	5	TAGTAAGCCA	NC_009794	5457
pEC278	P	5	TTAAGCCAGT	AY589571	824
pSW200	P	5	TTAAGCCAGT	L42525	1175
pUCD5000	P	5	AGAAGCCAGT	NC_001898	1502
pEC01	P	5	GTAAGCCAGT	AB117929	162
pTcM1	Q	1	CAGCCTCCGC	EU421841	25051
pMG828-4	Q	1	GGAAGCGCAC	NC_008489	5418
pDOJH10L	Q	u	TAGGGCGCAC	NC_004252	7384
pRL12	Q	u	ATGCCGGTTA	NC_008378	303172
pB44	Q	u	TAGGGCGCAC	NC_004443	3507
pMG1	Q	u	TAGGGCGCAC	NC_006997	3566
pSK41	Q	u	GTAAGGGCGC	NC_005024	10221
pLW043	Q	u	GTAAGGGCGC	NC_005054	55006
pTB6	Q	u	TAGGGCGCAC	NC_006843	3503
pUSA03	Q	u	GTAAGGGCGC	NC_007792	34253
pSMED02	Q	2	AAGGGCGCAA	NC_009621	359692
pMS260	Q	1	AGGGCGCACT	NC_005312	3957
pVM111	Q	1	AGGGCGCACT	AJ514834	5916
pIE1130	Q	1	AGGGCGCACT	NC_004973	820
pIE1115	Q	1	GAGGGCGCAC	NC_002524	377
pCCK381	Q	1	AGGGCGCACT	NC_006994	869
DN1	Q	1	GAGGGCGCAC	NC_002636	861
pCAUL02	Q	u	CAGCGGCTGG	NC_010333	82716
p11745	Q	1	TAGTGCCCAC	DQ176855	757
pIGJC156	Q	1	GGAAGCGCAC	NC_009781	2658
PNAC2	Q	u	TAGGGCGCAC	NC_004769	1085
p6043B	Q	u	TAGGGCGCAC	DQ458911	195
pRL8	Q	2	AGGGCGCAAT	NC_008383	126432
p42d	Q	2	GAGGGCGCAA	NC_004041	182616
pAt	Q	2	GAGGGCGCAA	NC_003064	116136
pRi2659	Q	2	AAGGGCGCAA	EU186381	106649
pRi1724	Q	2	AAGGGCGCAA	NC_002575	140060
pXAUT01	Q	2	GAGGGCGCAA	NC_009717	220687
pRiA4b	Q	2	AAGGGCGCAA	AB050904	9994
pRL7	Q	2	AAGGGCGCAA	NC_008382	69454
pTiBo542	Q	2	AGGGTCCAAG	DQ058764	213656
pSmeSM11b	Q	2	AGGGCGCAAT	EF066650	157471
pTi-SAKURA	Q	2	AGGGCGCAAT	NC_002147	100335
pHCG3	Q	2	GGGCGCAGTA	NC_005873	110160
pTi	Q	2	AGGGCGCAAT	DQ195264	1760
pTi	Q	2	AGGGCGCAAT	DQ195264	30727
pTi	Q	2	GGGCGCAATT	DQ195264	143879
pSSU1	V	1	TATAATAATA	NC_002140	2429
pSMQ172	V	1	TATAACGCAC	AF295100	1359
pER13	V	1	GTATAACGCA	NC_002776	1864
pPB1	V	1	TATAACCCAC	NC_006399	1660
pLB4	V	1	TATAACCCAC	M33531	526
pYSI8	V	u	TATAACACAC	EU185047	3630
pSMA23	V	1	TATAGCCCAA	NC_010242	545
pLC88	V	1	TATAGCCCAA	U31333	2075
pBS608	V	1	TGTAACGCAC	NC_006825	3563
pTA1015	V	1	TGTAACGCAC	NC_001765	2772
p1414	V	1	TGTAACGCAC	NC_002075	4365
pSES22	V	1	ATAACACACT	NC_007621	2572
pTB19	V	1	TATAGCACAC	M63891	9895
pTB53	V	1	ATAACACACT	D14852	424
pTB913	V	1	TATAGCACAC	M63891	9895
pRS3	V	u	ACTAACTTGC	NC_003099	141
pRW35	V	1	ATACACACTA	EU192194	4551
pAMalpha1	V	1	ATAACACACT	NC_005013	8466
pK214	V	1	TATAACCCAC	X92946	21706
pBC16	V	1	ATAACACACT	NC_001705	1155
pUB110	V	1	ATAACACACT	NC_001384	1155
pCCK381	V	2	GTGTACTCAC	NC_006994	5223
pC	V	5	CGGAAGGCGC	NC_007489	88866

Table S3. *P* values of pairwise *F* test (*t* test) between MOB groups.

	MOB P	MOB Q	MOB V
MOB F	< 0.001	0,050	< 0.001
MOB P		0,006	< 0.001
MOB Q			< 0.001

Table S4. Ranking of structural variables using machine learning algorithms. Variables were ranked according to relative importance based on 10 repetitions of 10-fold cross validations using CSF and ReliefF algorithms.

	Csf algorithm				ReliefF algorithm			
Ranking	Average number of folds	STD	Variable no.	Variable name	Average merit	STD	Variable no.	Variable name
1	10	0,000	123	S_Def_13	0,186	,000422	123	S_Def_13
2	10	0,000	79	S_Stab_13	0,163	,000000	76	S_Stab_10
3	10	0,000	34	S_Per_12	0,155	,000527	80	S_Stab_14
4	10	0,000	14	S_Bend_14	0,152	,000316	14	S_Bend_14
5	9,9	0,316	80	S_Stab_14	0,151	,000000	124	S_Def_14
6	9,7	0,483	101	S_TIDD_13	0,139	,000516	98	S_TIDD_10
7	9,7	0,675	36	S_Per_14	0,138	,000422	122	S_Def_12
8	9,6	0,516	127	S_Def_17	0,135	,000483	117	S_Def_7
9	9,6	0,699	76	S_Stab_10	0,133	,000000	120	S_Def_10
10	8,6	0,966	102	S_TIDD_14	0,130	,000316	10	S_Bend_10
11	8,4	0,843	122	S_Def_12	0,130	,000316	34	S_Per_12
12	8,2	0,632	73	S_Stab_7	0,127	,000422	79	S_Stab_13
13	6,9	0,994	98	S_TIDD_10	0,127	,000516	13	S_Bend_13
14	6,8	0,919	103	S_TIDD_15	0,122	,000422	73	S_Stab_7
15	6,4	0,966	5	S_Bend_5	0,122	,000471	78	S_Stab_12
16	6,2	2,201	124	S_Def_14	0,120	,000000	127	S_Def_17
17	6,1	1,197	118	S_Def_8	0,119	,000483	56	S_Hel_12
18	5,3	1,337	46	S_Hel_2	0,111	,000422	75	S_Stab_9
19	5,2	2,098	58	S_Hel_14	0,107	,000483	71	S_Stab_5
20	4,8	1,687	105	S_TIDD_17	0,106	,000483	36	S_Per_14
21	4,3	1,160	2	S_Bend_2	0,104	,000483	125	S_Def_15
22	4,2	1,317	72	S_Stab_6	0,102	,000316	72	S_Stab_6
23	4	1,333	74	S_Stab_8	0,099	,000483	81	S_Stab_15
24	3,9	1,729	120	S_Def_10	0,099	,000516	119	S_Def_9
25	3,9	1,524	83	S_Stab_17	0,097	,000516	5	S_Bend_5
26	3,9	1,197	63	S_Hel_19	0,096	,000000	83	S_Stab_17
27	3,8	1,033	96	S_TIDD_8	0,096	,000000	39	S_Per_17
28	3,7	1,160	93	S_TIDD_5	0,095	,000568	93	S_TIDD_5
29	3,5	0,707	24	S_Per_2	0,095	,000471	11	S_Bend_11
30	3,4	1,430	107	S_TIDD_19	0,095	,000675	103	S_TIDD_15
31	3	1,155	71	S_Stab_5	0,094	,000516	77	S_Stab_11
32	2,7	1,889	85	S_Stab_19	0,093	,000471	100	S_TIDD_12
33	2,7	0,675	53	S_Hel_9	0,091	,000000	101	S_TIDD_13
34	2,6	1,265	28	S_Per_6	0,090	,000316	61	S_Hel_17
35	2,6	1,265	10	S_Bend_10	0,089	,000516	52	S_Hel_8
36	2,5	1,080	56	S_Hel_12	0,089	,000527	57	S_Hel_13
37	2,5	0,850	25	S_Per_3	0,086	,000000	116	S_Def_6
38	2,4	1,075	125	S_Def_15	0,086	,000000	54	S_Hel_10
39	2,4	1,265	48	S_Hel_4	0,086	,000000	8	S_Bend_8
40	2,2	0,632	99	S_TIDD_11	0,086	,000516	55	S_Hel_11
41	2,2	1,033	94	S_TIDD_6	0,085	,000000	74	S_Stab_8
42	2	0,816	78	S_Stab_12	0,084	,000000	113	S_Def_3
43	1,8	0,919	109	S_TIDD_21	0,084	,000516	118	S_Def_8
44	1,7	0,823	67	S_Stab_1	0,084	,000527	51	S_Hel_7
45	1,7	1,059	30	S_Per_8	0,083	,000316	65	S_Hel_21
46	1,5	1,179	100	S_TIDD_12	0,083	,000316	50	S_Hel_6
47	1,3	0,675	27	S_Per_5	0,083	,000000	12	S_Bend_12
48	1,2	0,789	47	S_Hel_3	0,082	,000568	95	S_TIDD_7
49	1,1	0,738	106	S_TIDD_18	0,081	,000699	33	S_Per_11
50	1,1	1,197	32	S_Per_10	0,080	,000000	94	S_TIDD_6
51	1	1,054	65	S_Hel_21	0,080	,000000	82	S_Stab_16
52	1	1,054	52	S_Hel_8	0,079	,000000	69	S_Stab_3
53	1	0,943	16	S_Bend_16	0,078	,000316	27	S_Per_5
54	1	0,816	13	S_Bend_13	0,077	,000675	19	S_Bend_19
55	0,9	0,738	110	S_TIDD_22	0,077	,000316	31	S_Per_9
56	0,9	0,876	40	S_Per_18	0,076	,000316	25	S_Per_3
57	0,7	0,823	130	S_Def_20	0,075	,000316	47	S_Hel_3
58	0,6	0,516	112	S_Def_2	0,074	,000422	18	S_Bend_18
59	0,6	1,075	88	S_Stab_22	0,073	,000316	63	S_Hel_19
60	0,6	0,843	42	S_Per_20	0,073	,000568	6	S_Bend_6
61	0,6	0,699	20	S_Bend_20	0,072	,000422	32	S_Per_10
62	0,5	0,707	89	S_TIDD_1	0,072	,000000	70	S_Stab_4
63	0,5	0,527	82	S_Stab_16	0,072	,000483	130	S_Def_20
64	0,4	0,516	81	S_Stab_15	0,071	,000000	97	S_TIDD_9
65	0,4	0,516	55	S_Hel_11	0,071	,000316	115	S_Def_5
66	0,4	0,516	39	S_Per_17	0,071	,000316	3	S_Bend_3
67	0,4	0,516	35	S_Per_13	0,071	,000483	29	S_Per_7
68	0,4	0,516	7	S_Bend_7	0,070	,000516	99	S_TIDD_11
69	0,3	0,675	95	S_TIDD_7	0,070	,000949	28	S_Per_6
70	0,3	0,483	90	S_TIDD_2	0,070	,000000	91	S_TIDD_3
71	0,3	0,483	26	S_Per_4	0,070	,000422	58	S_Hel_14
72	0,3	0,483	9	S_Bend_9	0,069	,000516	104	S_TIDD_16
73	0,3	0,483	4	S_Bend_4	0,067	,000527	102	S_TIDD_14
74	0,3	0,483	1	S_Bend_1	0,066	,000422	132	S_Def_22
75	0,2	0,632	128	S_Def_18	0,066	,000527	126	S_Def_16
76	0,2	0,422	117	S_Def_7	0,064	,000422	49	S_Hel_5
77	0,2	0,422	68	S_Stab_2	0,064	,000632	121	S_Def_11
78	0,2	0,422	60	S_Hel_16	0,063	,000568	20	S_Bend_20
79	0,2	0,422	54	S_Hel_10	0,062	,000000	53	S_Hel_9
80	0,2	0,422	21	S_Bend_21	0,062	,000471	7	S_Bend_7
81	0,2	0,422	3	S_Bend_3	0,062	,000527	30	S_Per_8
82	0,1	0,316	126	S_Def_16	0,061	,000422	9	S_Bend_9
83	0,1	0,316	61	S_Hel_17	0,061	,000000	114	S_Def_4
84	0	0,000	132	S_Def_22	0,061	,000316	85	S_Stab_19
85	0	0,000	131	S_Def_21	0,061	,000422	88	S_Stab_22
86	0	0,000	129	S_Def_19	0,060	,000516	112	S_Def_2
87	0	0,000	121	S_Def_11	0,059	,000483	86	S_Stab_20
88	0	0,000	119	S_Def_9	0,059	,000316	96	S_TIDD_8
89	0	0,000	116	S_Def_6	0,058	,000316	24	S_Per_2
90	0	0,000	115	S_Def_5	0,058	,000471	68	S_Stab_2
91	0	0,000	114	S_Def_4	0,057	,000483	15	S_Bend_15
92	0	0,000	113	S_Def_3	0,057	,000516	2	S_Bend_2
93	0	0,000	111	S_Def_1	0,056	,000316	44	S_Per_22
94	0	0,000	108	S_TIDD_20	0,056	,000316	108	S_TIDD_20
95	0	0,000	104	S_TIDD_16	0,056	,000568	60	S_Hel_16
96	0	0,000	97	S_TIDD_9	0,056	,000483	43	S_Per_21
97	0	0,000	92	S_TIDD_4	0,054	,000422	22	S_Bend_22
98	0	0,000	91	S_TIDD_3	0,054	,000316	26	S_Per_4
99	0	0,000	87	S_Stab_21	0,054	,000422	105	S_TIDD_17
100	0	0,000	86	S_Stab_20	0,054	,000422	4	S_Bend_4
101	0	0,000	84	S_Stab_18	0,053	,000422	128	S_Def_18
102	0	0,000	77	S_Stab_11	0,053	,000316	17	S_Bend_17
103	0	0,000	75	S_Stab_9	0,053	,000422	84	S_Stab_18
104	0	0,000	70	S_Stab_4	0,053	,000527	21	S_Bend_21
105	0	0,000	69	S_Stab_3	0,052	,000422	111	S_Def_1
106	0	0,000	66	S_Hel_22	0,052	,000527	67	S_Stab_1
107	0	0,000	64	S_Hel_20	0,051	,000422	48	S_Hel_4
108	0	0,000	62	S_Hel_18	0,051	,000471	35	S_Per_13
109	0	0,000	59	S_Hel_15	0,050	,000316	38	S_Per_16
110	0	0,000	57	S_Hel_13	0,050	,000000	46	S_Hel_2
111	0	0,000	51	S_Hel_7	0,048	,000316	92	S_TIDD_4
112	0	0,000	50	S_Hel_6	0,048	,000000	16	S_Bend_16
113	0	0,000	49	S_Hel_5	0,048	,000422	129	S_Def_19
114	0	0,000	45	S_Hel_1	0,048	,000699	131	S_Def_21
115	0	0,000	44	S_Per_22	0,046	,000316	87	S_Stab_21
116	0	0,000	43	S_Per_21	0,046	,000316	62	S_Hel_18
117	0	0,000	41	S_Per_19	0,046	,000316	37	S_Per_15
118	0	0,000	38	S_Per_16	0,044	,000000	59	S_Hel_15
119	0	0,000	37	S_Per_15	0,043	,000422	66	S_Hel_22
120	0	0,000	33	S_Per_11	0,043	,000316	42	S_Per_20
121	0	0,000	31	S_Per_9	0,040	,000483	40	S_Per_18
122	0	0,000	29	S_Per_7	0,040	,000316	90	S_TIDD_2
123	0	0,000	23	S_Per_1	0,039	,000316	110	S_TIDD_22
124	0	0,000	22	S_Bend_22	0,036	,000316	107	S_TIDD_19
125	0	0,000	19	S_Bend_19	0,035	,000516	1	S_Bend_1
126	0	0,000	18	S_Bend_18	0,035	,000000	109	S_TIDD_21
127	0	0,000	17	S_Bend_17	0,035	,000000	89	S_TIDD_1
128	0	0,000	15	S_Bend_15	0,035	,000483	23	S_Per_1
129	0	0,000	12	S_Bend_12	0,033	,000422	106	S_TIDD_18
130	0	0,000	11	S_Bend_11	0,031	,000516	45	S_Hel_1
131	0	0,000	8	S_Bend_8	0,031	,000483	64	S_Hel_20
132	0	0,000	6	S_Bend_6	0,027	,000000	41	S_Per_19

Table S5. Evaluation of best variable subsets using different variable rankings. Best subsets were selected based on results of 10 repetitions of testing models that were trained on 64 elements with the set of 136 elements.

		Classification accuracy		Kappa statistic		Precision		Recall		Matthews CC		Area under ROC curve	
Ranking method	Subset	Mean	STD	Mean	STD	Mean	STD	Mean	STD	Mean	STD	Mean	STD
none	132 variables	0,919	0,013	0,885	0,017	0,927	0,014	0,919	0,013	0,890	0,018	0,988	0,011
ReliefF	16 variables	0,975	0,029	0,964	0,038	0,976	0,032	0,975	0,029	0,967	0,039	0,994	0,006
CFS	20 variables	0,956	0,012	0,936	0,017	0,958	0,012	0,956	0,012	0,938	0,016	0,988	0,007
P value ranking	24 variables	0,919	0,025	0,886	0,033	0,931	0,024	0,919	0,025	0,887	0,033	0,928	0,010

Table S6. Classification tests using the subset of 16 highest ranked variables. Subsets of elements that were the most frequently inaccurately classified (low classification frequency, CF) were removed to test the effect that this had on construction of predictive models. The classification tests comprised (i) 10-fold cross validations using the training dataset (CV_64), (ii) testing the trained models with the testing dataset (Test) and (iii) 10-fold CVs using the full set of 200 elements (CV_200). Standard deviations (STD) of the classification results are given.

Element	Classification frequency (CF)	MOB group	MOB subgroup	Possible cause for low CF	Removed subset based on CF cutoff
					A
pNL1	0	F	1	unique element in subgroup	B (=0)
pTi-VirD2(RB)	0	P	2	unique element	
pC221	0	P	7	unique element	
pIP501	0	Q	3	unique element	
pKJ36	0	Q	u	subgroup unknown	
pTA1060	0	V	1	/	
Tn4451	0	V	u	subgroup unknown	
pWW0	0,03	F	11	separate cluster from elements in subgroup	C (<0,1)
ColE1	0,13	P	5	unique element	D (<0,2)
pIP421	0,25	V	4	/	E(<0,3)
pAB6	0,34	Q	1	separate cluster from elements in subgroup	F(<0,5)
R6K	0,39	P	3	unique element	

Removed subset based on CF cutoff	Average classification accuracy					
	CV_64	STD	Test	STD	CV_200	STD
A	0,790	0,030	0,974	0,004	0,949	0,008
B (=0)	0,936	0,026	0,871	0,000	0,964	0,006
C (<0,1)	0,941	0,024	0,881	0,005	0,959	0,008
D (<0,2)	0,979	0,018	0,764	0,002	0,960	0,006
E(<0,3)	0,977	0,020	0,819	0,006	0,965	0,006
F(<0,5)	0,960	0,013	0,853	0,006	0,965	0,006

Removed subset based on CF cutoff	Kappa statistic					
	CV_64	STD	Test	STD	CV_200	STD
A	0,692	0,044	0,962	0,006	0,926	0,012
B (=0)	0,900	0,042	0,813	0,000	0,948	0,009
C (<0,1)	0,909	0,038	0,823	0,007	0,941	0,012
D (<0,2)	0,967	0,028	0,666	0,003	0,942	0,009
E(<0,3)	0,965	0,033	0,738	0,008	0,949	0,009
F(<0,5)	0,937	0,023	0,785	0,009	0,950	0,009

Removed subset based on CF cutoff	Precision (*Pre*)					
	CV_64	STD	Test	STD	CV_200	STD
A	0,842	0,041	0,975	0,004	0,958	0,007
B (=0)	0,953	0,027	0,875	0,000	0,970	0,005
C (<0,1)	0,959	0,025	0,893	0,006	0,967	0,007
D (<0,2)	0,988	0,016	0,789	0,003	0,967	0,006
E(<0,3)	0,990	0,014	0,826	0,004	0,972	0,005
F(<0,5)	0,970	0,021	0,858	0,006	0,972	0,005

Contunued below…

Continued from above…

Removed subset based on CF cutoff	Recall (*Rec*)					
	CV_64	STD	Test	STD	CV_200	STD
A	0,790	0,030	0,973	0,005	0,949	0,008
B (=0)	0,935	0,026	0,871	0,000	0,964	0,006
C (<0,1)	0,941	0,024	0,881	0,005	0,959	0,008
D (<0,2)	0,979	0,018	0,763	0,002	0,960	0,006
E(<0,3)	0,978	0,019	0,818	0,006	0,965	0,006
F(<0,5)	0,960	0,014	0,853	0,006	0,965	0,006

Removed subset based on CF cutoff	Matthews correlation coefficient					
	CV_64	STD	Test	STD	CV_200	STD
A	0,728	0,045	0,965	0,005	0,931	0,011
B (=0)	0,915	0,036	0,806	0,000	0,951	0,009
C (<0,1)	0,923	0,033	0,825	0,008	0,945	0,011
D (<0,2)	0,971	0,029	0,661	0,004	0,946	0,008
E(<0,3)	0,972	0,028	0,731	0,008	0,954	0,009
F(<0,5)	0,947	0,021	0,780	0,009	0,954	0,008

Removed subset based on CF cutoff	Area under ROC curve					
	CV_64	STD	Test	STD	CV_200	STD
A	0,924	0,017	0,994	0,001	0,986	0,003
B (=0)	0,992	0,007	0,949	0,001	0,990	0,002
C (<0,1)	0,997	0,011	0,930	0,002	0,990	0,003
D (<0,2)	0,998	0,014	0,934	0,002	0,990	0,002
E(<0,3)	0,998	0,014	0,932	0,001	0,991	0,002
F(<0,5)	0,999	0,003	0,927	0,002	0,991	0,002

Table S7. Ranges of potential transfer hosts based on pooling known transfer host clades found in the training sets of either 64 or 200 elements.

	MOB group	Potential transfer host clades			
		Species	Order	Class	Phylum
Set of 64 elements	F	*Escherichia coli*	*Enterobacteriales*	*Gammaproteobacteria*	*Proteobacteria*
		Nostoc sp.	*Nostocales*	-	*Cyanobacteria*
		Novosphingobium aromaticivorans	*Sphingomonadales*	*Alphaproteobacteria*	*Proteobacteria*
		Pseudomonas putida	*Pseudomonadales*	*Gammaproteobacteria*	*Proteobacteria*
		Pseudomonas sp.	*Pseudomonadales*	*Gammaproteobacteria*	*Proteobacteria*
		Salmonella enterica	*Enterobacteriales*	*Gammaproteobacteria*	*Proteobacteria*
		Salmonella typhi	*Enterobacteriales*	*Gammaproteobacteria*	*Proteobacteria*
		Salmonella typhimurium	*Enterobacteriales*	*Gammaproteobacteria*	*Proteobacteria*
		Shigella flexneri	*Enterobacteriales*	*Gammaproteobacteria*	*Proteobacteria*
	P	*Acidithiobacillus caldus*	*Acidithiobacillales*	*Gammaproteobacteria*	*Proteobacteria*
		Acidithiobacillus ferrooxidans	*Acidithiobacillales*	*Gammaproteobacteria*	*Proteobacteria*
		Agrobacterium tumefaciens	*Rhizobiales*	*Alphaproteobacteria*	*Proteobacteria*
		Collimonas fungivorans	*Burkholderiales*	*Betaproteobacteria*	*Proteobacteria*
		Enterobacter aerogenes	*Enterobacteriales*	*Gammaproteobacteria*	*Proteobacteria*
		Escherichia coli	*Enterobacteriales*	*Gammaproteobacteria*	*Proteobacteria*
		Pseudomonas aeruginosa	*Pseudomonadales*	*Gammaproteobacteria*	*Proteobacteria*
		Pseudomonas alcaligenes	*Pseudomonadales*	*Gammaproteobacteria*	*Proteobacteria*
		Salmonella typhimurium	*Enterobacteriales*	*Gammaproteobacteria*	*Proteobacteria*
		Staphylococcus aureus	*Bacillales*	*Bacilli*	*Firmicutes*
		Vibrio fischeri	*Vibrionales*	*Gammaproteobacteria*	*Proteobacteria*
		Xylella fastidiosa	*Xanthomonadales*	*Gammaproteobacteria*	*Proteobacteria*
	Q	*Acidithiobacillus ferrooxidans*	*Acidithiobacillales*	*Gammaproteobacteria*	*Proteobacteria*
		Agrobacterium tumefaciens	*Rhizobiales*	*Alphaproteobacteria*	*Proteobacteria*
		Bifidobacterium longum	*Bifidobacteriales*	*Actinobacteria*	*Actinobacteria*
		Dichelobacter nodosus	*Cardiobacteriales*	*Gammaproteobacteria*	*Proteobacteria*
		Escherichia coli	*Enterobacteriales*	*Gammaproteobacteria*	*Proteobacteria*
		Mesorhizobium sp.	*Rhizobiales*	*Alphaproteobacteria*	*Proteobacteria*
		Neisseria meningitidis	*Neisseriales*	*Betaproteobacteria*	*Proteobacteria*
		Rhizobium etli	*Rhizobiales*	*Alphaproteobacteria*	*Proteobacteria*
		Rhizobium sp.	*Rhizobiales*	*Alphaproteobacteria*	*Proteobacteria*
		Rhodobacter blasticus	*Rhodobacterales*	*Alphaproteobacteria*	*Proteobacteria*
		Salmonella enteritidis	*Enterobacteriales*	*Gammaproteobacteria*	*Proteobacteria*
		Salmonella typhimurium	*Enterobacteriales*	*Gammaproteobacteria*	*Proteobacteria*
		Sinorhizobium meliloti	*Planctomycetales*	*Planctomycetia*	*Planctomycetes*
		Staphylococcus aureus	*Bacillales*	*Bacilli*	*Firmicutes*
		Streptococcus	*Lactobacillales*	*Bacilli*	*Firmicutes*
	V	*Bacillus subtilis*	*Bacillales*	*Bacilli*	*Firmicutes*
		Bacteroides fragilis	*Bacteroidales*	*Bacteroidia*	*Bacteroidetes*
		Bacteroides uniformis	*Bacteroidales*	*Bacteroidia*	*Bacteroidetes*
		Bordetella bronchiseptica	*Burkholderiales*	*Betaproteobacteria*	*Proteobacteria*
		Butyrivibrio fibrisolvens	*Clostridiales*	*Clostridia*	*Firmicutes*
		Clostridium perfringens	*Clostridiales*	*Clostridia*	*Firmicutes*
		Flavobacterium sp.	*Flavobacteriales*	*Flavobacteriia*	*Bacteroidetes*
		Lactobacillus hilgardii	*Lactobacillales*	*Bacilli*	*Firmicutes*
		Listeria monocytogenes	*Bacillales*	*Bacilli*	*Firmicutes*
		Oenococcus oeni	*Lactobacillales*	*Bacilli*	*Firmicutes*
		Staphylococcus cohnii	*Bacillales*	*Bacilli*	*Firmicutes*
		Streptococcus agalactiae	*Lactobacillales*	*Bacilli*	*Firmicutes*
		Streptococcus ferus	*Lactobacillales*	*Bacilli*	*Firmicutes*
		Treponema denticola	*Spirochaetales*	*Spirochaetia*	*Spirochaetes*

	MOB group	Potential transfer host clades			
		Species	Order	Class	Phylum
Set of 200 elements	F	*Escherichia coli*	*Enterobacteriales*	*Gammaproteobacteria*	*Proteobacteria*
		Klebsiella pneumoniae	*Enterobacteriales*	*Gammaproteobacteria*	*Proteobacteria*
		Nostoc sp.	*Nostocales*	-	*Cyanobacteria*
		Novosphingobium aromaticivorans	*Sphingomonadales*	*Alphaproteobacteria*	*Proteobacteria*
		Pseudomonas putida	*Pseudomonadales*	*Gammaproteobacteria*	*Proteobacteria*
		Pseudomonas sp.	*Pseudomonadales*	*Gammaproteobacteria*	*Proteobacteria*
		Rhodococcus erythropolis	*Actinomycetales*	*Actinobacteria*	*Actinobacteria*
		Salmonella enterica	*Enterobacteriales*	*Gammaproteobacteria*	*Proteobacteria*
		Salmonella typhi	*Enterobacteriales*	*Gammaproteobacteria*	*Proteobacteria*
		Salmonella typhimurium	*Enterobacteriales*	*Gammaproteobacteria*	*Proteobacteria*
		Shigella flexneri	*Enterobacteriales*	*Gammaproteobacteria*	*Proteobacteria*
	P	*Achromobacter denitrificans*	*Burkholderiales*	*Betaproteobacteria*	*Proteobacteria*
		Achromobacter xylosoxidans	*Burkholderiales*	*Betaproteobacteria*	*Proteobacteria*
		Acidithiobacillus caldus	*Acidithiobacillales*	*Gammaproteobacteria*	*Proteobacteria*
		Acidithiobacillus ferrooxidans	*Acidithiobacillales*	*Gammaproteobacteria*	*Proteobacteria*
		Acidovorax sp.	*Burkholderiales*	*Betaproteobacteria*	*Proteobacteria*
		Agrobacterium tumefaciens	*Rhizobiales*	*Alphaproteobacteria*	*Proteobacteria*
		Bordetella pertussis	*Burkholderiales*	*Betaproteobacteria*	*Proteobacteria*
		Citrobacter koseri	*Enterobacteriales*	*Gammaproteobacteria*	*Proteobacteria*
		Collimonas fungivorans	*Burkholderiales*	*Betaproteobacteria*	*Proteobacteria*
		Delftia acidovorans	*Burkholderiales*	*Betaproteobacteria*	*Proteobacteria*
		Enterobacter aerogenes	*Enterobacteriales*	*Gammaproteobacteria*	*Proteobacteria*
		Enterobacter cloacae	*Enterobacteriales*	*Gammaproteobacteria*	*Proteobacteria*
		Enterococcus faecium	*Lactobacillales*	*Bacilli*	*Firmicutes*
		Erwinia stewartii	*Enterobacteriales*	*Gammaproteobacteria*	*Proteobacteria*
		Erwinia tasmaniensis	*Enterobacteriales*	*Gammaproteobacteria*	*Proteobacteria*
		Escherichia coli	*Enterobacteriales*	*Gammaproteobacteria*	*Proteobacteria*
		Hafnia alvei	*Enterobacteriales*	*Gammaproteobacteria*	*Proteobacteria*
		Lactobacillus brevis	*Lactobacillales*	*Bacilli*	*Firmicutes*
		Mesorhizobium sp.	*Rhizobiales*	*Alphaproteobacteria*	*Proteobacteria*
		Pantoea citrea	*Enterobacteriales*	*Gammaproteobacteria*	*Proteobacteria*
		Pseudomonas aeruginosa	*Pseudomonadales*	*Gammaproteobacteria*	*Proteobacteria*
		Pseudomonas alcaligenes	*Pseudomonadales*	*Gammaproteobacteria*	*Proteobacteria*
		Pseudomonas sp.	*Pseudomonadales*	*Gammaproteobacteria*	*Proteobacteria*
		Ralstonia eutropha	*Burkholderiales*	*Betaproteobacteria*	*Proteobacteria*
		Rhodobacter sphaeroides	*Rhodobacterales*	*Alphaproteobacteria*	*Proteobacteria*
		Salmonella choleraesuis	*Enterobacteriales*	*Gammaproteobacteria*	*Proteobacteria*
		Salmonella enterica serovar Borreze	*Enterobacteriales*	*Gammaproteobacteria*	*Proteobacteria*
		Salmonella enterica subsp. enterica	*Enterobacteriales*	*Gammaproteobacteria*	*Proteobacteria*
		Salmonella enteritidis	*Enterobacteriales*	*Gammaproteobacteria*	*Proteobacteria*
		Salmonella typhimurium	*Enterobacteriales*	*Gammaproteobacteria*	*Proteobacteria*
		Shigella sonnei	*Enterobacteriales*	*Gammaproteobacteria*	*Proteobacteria*
		Sphingomonas sp.	*Sphingomonadales*	*Alphaproteobacteria*	*Proteobacteria*
		Staphylococcus aureus	*Bacillales*	*Bacilli*	*Firmicutes*
		Staphylococcus epidermidis	*Bacillales*	*Bacilli*	*Firmicutes*
		Staphylococcus haemolyticus	*Bacillales*	*Bacilli*	*Firmicutes*
		Vibrio fischeri	*Vibrionales*	*Gammaproteobacteria*	*Proteobacteria*
		Xylella fastidiosa	*Xanthomonadales*	*Gammaproteobacteria*	*Proteobacteria*
		Xylella fastidiosa	*Xanthomonadales*	*Gammaproteobacteria*	*Proteobacteria*
		Yersinia pseudotuberculosis	*Enterobacteriales*	*Gammaproteobacteria*	*Proteobacteria*
	Q	*Acidithiobacillus caldus*	*Acidithiobacillales*	*Gammaproteobacteria*	*Proteobacteria*
		Acidithiobacillus ferrooxidans	*Acidithiobacillales*	*Gammaproteobacteria*	*Proteobacteria*
		Actinobacillus pleuropneumoniae	*Pasteurellales*	*Gammaproteobacteria*	*Proteobacteria*
		Agrobacterium rhizogenes	*Rhizobiales*	*Alphaproteobacteria*	*Proteobacteria*
		Agrobacterium tumefaciens	*Rhizobiales*	*Alphaproteobacteria*	*Proteobacteria*
		Bifidobacterium longum	*Bifidobacteriales*	*Actinobacteria*	*Actinobacteria*
		Caulobacter sp.	*Caulobacterales*	*Alphaproteobacteria*	*Proteobacteria*
		Dichelobacter nodosus	*Cardiobacteriales*	*Gammaproteobacteria*	*Proteobacteria*
		Escherichia coli	*Enterobacteriales*	*Gammaproteobacteria*	*Proteobacteria*
		Mesorhizobium sp.	*Rhizobiales*	*Alphaproteobacteria*	*Proteobacteria*
		Neisseria meningitidis	*Neisseriales*	*Betaproteobacteria*	*Proteobacteria*
		Oligotropha carboxidovorans	*Rhizobiales*	*Alphaproteobacteria*	*Proteobacteria*
		Pasteurella multocida	*Pasteurellales*	*Gammaproteobacteria*	*Proteobacteria*
		Rhizobium etli	*Rhizobiales*	*Alphaproteobacteria*	*Proteobacteria*
		Rhizobium leguminosarum	*Rhizobiales*	*Alphaproteobacteria*	*Proteobacteria*
		Rhizobium sp.	*Rhizobiales*	*Alphaproteobacteria*	*Proteobacteria*
		Rhodobacter blasticus	*Rhodobacterales*	*Alphaproteobacteria*	*Proteobacteria*
		Salmonella enteritidis	*Enterobacteriales*	*Gammaproteobacteria*	*Proteobacteria*
		Salmonella typhimurium	*Enterobacteriales*	*Gammaproteobacteria*	*Proteobacteria*
		Sinorhizobium medicae	*Planctomycetales*	*Planctomycetia*	*Planctomycetes*
		Sinorhizobium meliloti	*Planctomycetales*	*Planctomycetia*	*Planctomycetes*
		Staphylococcus aureus	*Bacillales*	*Bacilli*	*Firmicutes*
		Streptococcus	*Lactobacillales*	*Bacilli*	*Firmicutes*
		Xanthobacter autotrophicus	*Rhizobiales*	*Alphaproteobacteria*	*Proteobacteria*
	V	*Bacillus cereus*	*Bacillales*	*Bacilli*	*Firmicutes*
		Bacillus subtilis	*Bacillales*	*Bacilli*	*Firmicutes*
		Bacteroides fragilis	*Bacteroidales*	*Bacteroidia*	*Bacteroidetes*
		Bacteroides uniformis	*Bacteroidales*	*Bacteroidia*	*Bacteroidetes*
		Bordetella bronchiseptica	*Burkholderiales*	*Betaproteobacteria*	*Proteobacteria*
		Butyrivibrio fibrisolvens	*Clostridiales*	*Clostridia*	*Firmicutes*
		Clostridium perfringens	*Clostridiales*	*Clostridia*	*Firmicutes*
		Enterococcus faecalis	*Lactobacillales*	*Bacilli*	*Firmicutes*
		Flavobacterium sp.	*Flavobacteriales*	*Flavobacteriia*	*Bacteroidetes*
		Geobacillus stearothermophilus	*Bacillales*	*Bacilli*	*Firmicutes*
		Lactobacillus casei	*Lactobacillales*	*Bacilli*	*Firmicutes*
		Lactobacillus hilgardii	*Lactobacillales*	*Bacilli*	*Firmicutes*
		Lactobacillus plantarum	*Lactobacillales*	*Bacilli*	*Firmicutes*
		Lactobacillus sakei	*Lactobacillales*	*Bacilli*	*Firmicutes*
		Lactococcus lactis	*Lactobacillales*	*Bacilli*	*Firmicutes*
		Listeria monocytogenes	*Bacillales*	*Bacilli*	*Firmicutes*
		Oenococcus oeni	*Lactobacillales*	*Bacilli*	*Firmicutes*
		Pasteurella multocida	*Pasteurellales*	*Gammaproteobacteria*	*Proteobacteria*
		Rhodobacter sphaeroides	*Rhodobacterales*	*Alphaproteobacteria*	*Proteobacteria*
		Staphylococcus aureus	*Bacillales*	*Bacilli*	*Firmicutes*
		Staphylococcus cohnii	*Bacillales*	*Bacilli*	*Firmicutes*
		Staphylococcus saprophyticus	*Bacillales*	*Bacilli*	*Firmicutes*
		Streptococcus agalactiae	*Lactobacillales*	*Bacilli*	*Firmicutes*
		Streptococcus ferus	*Lactobacillales*	*Bacilli*	*Firmicutes*
		Streptococcus pyogenes	*Lactobacillales*	*Bacilli*	*Firmicutes*
		Streptococcus suis	*Lactobacillales*	*Bacilli*	*Firmicutes*
		Streptococcus thermophilus	*Lactobacillales*	*Bacilli*	*Firmicutes*
		Treponema denticola	*Spirochaetales*	*Spirochaetia*	*Spirochaetes*

Table S8. Ranges of potential incopatibility and replication (Inc/Rep) types based on pooling known Inc/Rep types found in the training sets of either 64 or 200 elements.

Set	MOB group	Inc/Rep types
Set of 64 elements	F	IncFI
		IncFII
		IncFV
		IncN
		IncP-9
		IncW
		pNL1
	P	ColE
		Inc4
		IncB/O
		IncFII
		IncI-1alpha
		IncI2
		IncP-1
		IncP-4 (IncQ)
		IncX2
		PromA
		RepABC
	Q	ColE2/ColE3
		Inc13, Inc7
		IncP-4 (IncQ)
		RepABC
		pNAC2
		pSC101
	V	Inc11
		Inc13
Set of 200 elements	F	IncFI
		IncZ
		ColE2/E3
		pCD1
		IncFII
		IncFV
		IncN
		IncP-9
		IncW
		pCD, IncT/Phage P1
		pNL1
		pREC1
	P	ColE
		Inc10
		Inc14
		Inc4
		IncB/O
		IncFI
		IncFII
		IncG/P6
		IncI-1alpha
		IncI2
		IncK
		IncP-1alpha
		IncP-1beta
		IncP-4 (IncQ)
		IncP-6
		IncX1
		IncX2
		PromA
		RepABC
	Q	ColE2/ColE3
		Inc1
		Inc13
		Inc7
		IncP-4 (IncQ)
		RepABC
		pNAC2
		pKJ50
		pSC101
	V	Inc11
		Inc13
		Inc4
		IncQ

References

Abo, T., and Ohtsubo, E. (1995). Characterization of the functional sites in the oriT region involved in DNA transfer promoted by sex factor plasmid R100. J. Bacteriol. 177, 4350–4355.

Avila, P., Núñez, B., and de la Cruz, F. (1996). Plasmid R6K Contains Two Functional< i> oriTs</i> which can Assemble Simultaneously in Relaxosomes< i> in vivo</i>. J. Mol. Biol. 261, 135–143.

Bhattacharjee, M.K., and Meyer, R.J. (1991). A segment of a plasmid gene required for conjugal transfer encodes a site-specific, single-strand DNA endonuclease and ligase. Nucleic Acids Res. 19, 1129–1137.

Bramucci, M., Chen, M., and Nagarajan, V. (2006). Genetic organization of a plasmid from an industrial wastewater bioreactor. Appl. Microbiol. Biotechnol. 71, 67–74.

Caryl, J.A., Smith, M.C., and Thomas, C.D. (2004). Reconstitution of a staphylococcal plasmid-protein relaxation complex in vitro. J. Bacteriol. 186, 3374–3383.

Chu, C., Chiu, C.-H., Chu, C.-H., and Ou, J.T. (2002). Nucleotide and amino acid sequences of oriT-traM-traJ-traY-traA-traL regions and mobilization of virulence plasmids of Salmonella enterica serovars Enteritidis, Gallinarum-Pullorum, and Typhimurium. J. Bacteriol. 184, 2857–2862.

Climo, M.W., Sharma, V.K., and Archer, G.L. (1996). Identification and characterization of the origin of conjugative transfer (oriT) and a gene (nes) encoding a single-stranded endonuclease on the staphylococcal plasmid pGO1. J. Bacteriol. 178, 4975–4983.

Cook, D.M., and Farrand, S.K. (1992). The oriT region of the Agrobacterium tumefaciens Ti plasmid pTiC58 shares DNA sequence identity with the transfer origins of RSF1010 and RK2/RP4 and with T-region borders. J. Bacteriol. 174, 6238–6246.

Coupland, G.M., Brown, A.M., and Willetts, N.S. (1987). The origin of transfer (oriT) of the conjugative plasmid R46: characterization by deletion analysis and DNA sequencing. Mol. Gen. Genet. MGG 208, 219–225.

Drolet, M., Zanga, P., and Lau, P.C.K. (1990). The mobilization and origin of transfer regions of a Thiobacillus ferrooxidans plasmid: relatedness to plasmids RSF1010 and pSC101. Mol. Microbiol. 4, 1381–1391.

Dunn, A.K., Martin, M.O., and Stabb, E.V. (2005). Characterization of pES213, a small mobilizable plasmid from< i> Vibrio fischeri</i>. Plasmid 54, 114–134.

Farías, M.E., and Espinosa, M. (2000). Conjugal transfer of plasmid pMV158: uncoupling of the pMV158 origin of transfer from the mobilization gene mobM, and modulation of pMV158 transfer in Escherichia coli mediated by IncP plasmids. Microbiology 146, 2259–2265.

Finlay, B.B., Frost, L.S., and Paranchych, W. (1986). Origin of transfer of IncF plasmids and nucleotide sequences of the type II oriT, traM, and traY alleles from ColB4-K98 and the type IV traY allele from R100-1. J. Bacteriol. 168, 132–139.

Francia, M., Varsaki, A., Garcillán-Barcia, M.P., Latorre, A., Drainas, C., and Cruz, F. (2004). A classification scheme for mobilization regions of bacterial plasmids. FEMS Microbiol. Rev. 28, 79–100.

Furuya, N., Nisioka, T., and Komano, T. (1991). Nucleotide sequence and functions of the oriT operon in IncI1 plasmid R64. J. Bacteriol. 173, 2231–2237.

Greated, A., Lambertsen, L., Williams, P.A., and Thomas, C.M. (2002). Complete sequence of the

IncP−9 TOL plasmid pWW0 from Pseudomonas putida. Environ. Microbiol. 4, 856–871.

Guasch, A., Lucas, M., Moncalián, G., Cabezas, M., Pérez-Luque, R., Gomis-Rüth, F.X., de la Cruz, F., and Coll, M. (2003). Recognition and processing of the origin of transfer DNA by conjugative relaxase TrwC. Nat. Struct. Mol. Biol. 10, 1002–1010.

Laurenzio, L., Frost, L.S., Finlay, B.B., and Paranchych, W. (1991). Characterization of the oriT region of the IncFV plasmid pED208. Mol. Microbiol. 5, 1779–1790.

Llosa, M., Grandoso, G., and Cruz, F. de la (1995). Nicking activity of TrwC directed against the origin of transfer of the IncW plasmid R388. J. Mol. Biol. 246, 54–62.

Matson, S.W., and Morton, B.S. (1991). Escherichia coli DNA helicase I catalyzes a site-and strand-specific nicking reaction at the F plasmid oriT. J. Biol. Chem. 266, 16232–16237.

Mela, F., Fritsche, K., Boersma, H., Van Elsas, J.D., Bartels, D., Meyer, F., De Boer, W., Van Veen, J.A., and Leveau, J.H. (2008). Comparative genomics of the pIPO2/pSB102 family of environmental plasmids: sequence, evolution, and ecology of pTer331 isolated from Collimonas fungivorans Ter331. FEMS Microbiol. Ecol. 66, 45–62.

Pansegrau, W., Ziegelin, G., and Lanka, E. (1988). The origin of conjugative IncP plasmid transfer: interaction with plasmid-encoded products and the nucleotide sequence at the relaxation site. Biochim. Biophys. Acta BBA-Gene Struct. Expr. 951, 365–374.

Paterson, E.S., and Iyer, V.N. (1997). Localization of the nic site of IncN conjugative plasmid pCU1 through formation of a hybrid oriT. J. Bacteriol. 179, 5768–5776.

Pérez-Mendoza, M., Schumacher, C., Suárez-García, F., Almazán-Almazán, M.C., Domingo-García, M., López-Garzón, F.J., and Seaton, N.A. (2006). Analysis of the microporous texture of a glassy carbon by adsorption measurements and Monte Carlo simulation. Evolution with chemical and physical activation. Carbon 44, 638–645.

Rawlings, D.E., and Tietze, E. (2001). Comparative biology of IncQ and IncQ-like plasmids. Microbiol. Mol. Biol. Rev. 65, 481–496.

Reygers, U., Wessel, R., Müller, H., and Hoffmann-Berling, H. (1991). Endonuclease activity of Escherichia coli DNA helicase I directed against the transfer origin of the F factor. EMBO J. 10, 2689.

Rohrer, J., and Rawlings, D.E. (1992). Sequence analysis and characterization of the mobilization region of a broad-host-range plasmid, pTF-FC2, isolated from Thiobacillus ferrooxidans. J. Bacteriol. 174, 6230–6237.

Scherzinger, E., Lurz, R., Otto, S., and Dobrinski, B. (1992). In vitro cleavage of double-and single-stranded DNA by plasmid RSF1010-encoded mobilization proteins. Nucleic Acids Res. 20, 41–48.

Smith, C.J., and Parker, A.C. (1998). The Transfer Origin for BacteroidesMobilizable Transposon Tn4555 Is Related to a Plasmid Family from Gram-Positive Bacteria. J. Bacteriol. 180, 435–439.

Sut, M.V., Mihajlovic, S., Lang, S., Gruber, C.J., and Zechner, E.L. (2009). Protein and DNA effectors control the TraI conjugative helicase of plasmid R1. J. Bacteriol. 191, 6888–6899.

Szpirer, C.Y., Faelen, M., and Couturier, M. (2001). Mobilization function of the pBHR1 plasmid, a derivative of the broad-host-range plasmid pBBR1. J. Bacteriol. 183, 2101–2110.

Tauch, A., Schneiker, S., Selbitschka, W., Pühler, A., van Overbeek, L.S., Smalla, K., Thomas, C.M., Bailey, M.J., Forney, L.J., and Weightman, A. (2002). The complete nucleotide sequence and

environmental distribution of the cryptic, conjugative, broad-host-range plasmid pIPO2 isolated from bacteria of the wheat rhizosphere. Microbiology 148, 1637–1653.

Tun-Garrido, C., Bustos, P., González, V., and Brom, S. (2003). Conjugative transfer of p42a from Rhizobium etli CFN42, which is required for mobilization of the symbiotic plasmid, is regulated by quorum sensing. J. Bacteriol. 185, 1681–1692.

Tzfira, T., Li, J., Lacroix, B., and Citovsky, V. (2004). < i> Agrobacterium</i> T-DNA integration: molecules and models. TRENDS Genet. 20, 375–383.

Varsaki, A., Moncalián, G., del Pilar Garcillán-Barcia, M., Drainas, C., and de la Cruz, F. (2009). Analysis of ColE1 MbeC unveils an extended ribbon-helix-helix family of nicking accessory proteins. J. Bacteriol. 191, 1446–1455.

Vedantam, G., Knopf, S., and Hecht, D.W. (2006). Bacteroides fragilis mobilizable transposon Tn5520 requires a 71 base pair origin of transfer sequence and a single mobilization protein for relaxosome formation during conjugation. Mol. Microbiol. 59, 288–300.

Wang, A., and Macrina, F.L. (1995). Streptococcal plasmid pIP501 has a functional oriT site. J. Bacteriol. 177, 4199–4206.

Wang, J., Shoemaker, N.B., Wang, G.-R., and Salyers, A.A. (2000). Characterization of a Bacteroides mobilizable transposon, NBU2, which carries a functional lincomycin resistance gene. J. Bacteriol. 182, 3559–3571.
